# *Vitex negundo* L. Essential Oil: Odorant Binding Protein Efficiency Using Molecular Docking Approach and Studies of the Mosquito Repellent

**DOI:** 10.3390/insects12121061

**Published:** 2021-11-26

**Authors:** Bamidele Joseph Okoli, Zakari Ladan, Fanyana Mtunzi, Yayock Chigari Hosea

**Affiliations:** 1 Department of Chemical Sciences, Faculty of Science and Technology, Bingham University, New Karu 961105, Nasarawa State, Nigeria; 2 Department of Chemistry, Faculty of Science, Kaduna State University, Tafawa Balewa Way, Kaduna 800283, Kaduna State, Nigeria; zakari.ladan@kasu.edu.ng; 3 Institute of Chemical and Biotechnology, Vaal University of Technology, Southern Gauteng Science and Technology Park, Private Bag X021, Vanderbijlpark 1911, South Africa; fanyana@vut.ac.za; 4 Department of Biological Sciences, Faculty of Science, Kaduna State University, Tafawa Balewa Way, Kaduna 800283, Kaduna State, Nigeria; yayock.hosea@kasu.edu.ng

**Keywords:** mosquito, repellent, *V. negundo*, molecular docking, odorant binding proteins, ligand efficiency metric, essential oil, *Anopheles gambiae*

## Abstract

**Simple Summary:**

Malaria fever kills millions of people annually in the tropical and subtropical countries of Africa and Asia. Because there is no effective vaccine, malaria prevention is exclusively dependent on avoiding human-vector interaction. The interaction of *Vitex negundo* essential oil constituents with *Anopheles gambiae* Odorant Binding Proteins (OBP), as well as its compositional variation, repellent efficacy, and toxicity profile, are investigated in this work. The oils were subjected to GC-MS analysis, a mosquito behavioral test, OBP-ligand interactions, *Anopheles* species authentication, and toxicity profile. Docking protocol validation was achieved by redocking the co-crystallized ligands and root mean square deviation (RMSD) calculation. The oil yields and compositions are climate–soil dependent with ≈71.39% monoterpenes and ≈16.32% sesquiterpene. Optimal repellency is achieved at 15 min at ED_50_ 0.08–0.48% *v*/*v* while the RMSD was estimated to be within 0.24–1.35 Å. Strong affinities, −6.4 to −5.4 kcal/mol, were demonstrated by α-pinene, citronellal, linalool, and myrcene for OBP1, OBP7, OBP4, and OBP. respectively. The hydrophobic interactions involve Leu17, Cys35, ALA52, Leu73, Leu76, Ala88, Met91, Lys93, Trp114, Phe123, and Leu124 receptors on α-helixes 1–7 within the binding cavities, and may block the olfactory receptors resulting in disorientation. α-pinene, linalool, and myrcene are safe and suitable for use in the development of green and innovative repellents because their ligand efficiency metrics, ADME/tox, and repellency screening are all within the threshold values.

**Abstract:**

(1) Background: Malaria fever affects millions of people yearly in Africa and Asia’s tropical and subtropical areas. Because there is no effective vaccine, malaria prevention is solely dependent on avoiding human-vector interaction. (2) Aim: This study examines the interaction between the constituents of *Vitex negundo* essential oil and *Anopheles gambiae* Odorant Binding Proteins (OBP) as well as the compositional variation, repellent efficacy, and toxicity profile. (3) Methods: The oils were subjected to GC-MS and mosquito behavioral analysis. OBP–ligand interactions, *Anopheles* species authentication, and the toxicity profile were determined by molecular docking, PCR assay and in silico ADME/tox tool. Docking protocol validation was achieved by redocking the co-crystallized ligands into the protein binding pocket and root mean square deviation (RMSD) calculation. (4) Results: The oil yields and compositions are climate–soil dependent with ≈71.39% monoterpenes and ≈16.32% sesquiterpene. Optimal repellency is achieved at 15 min at ED_50_ 0.08–0.48% *v*/*v* while the RMSD was estimated to be within 0.24–1.35 Å. Strong affinities were demonstrated by α-pinene (−6.4 kcal/mol), citronellal (−5.5 kcal/mol), linalool (−5.4 kcal/mol), and myrcene (−5.8 kcal/mol) for OBP1, OBP7, OBP4, and OBP; respectively. The hydrophobic interactions involve Leu17 (α-helix 1), Cys35 (α-helix 2), ALA52 (α-helix 3), Leu73, Leu76 (α-helix 4), Ala88, Met91, Lys93, Trp114 (α-helix 5), Phe123 (α-helix 6), and Leu124 (α-helix 7) receptors within the binding cavities, and may cause blocking of the olfactory receptors resulting in disorientation. (5) Conclusion: The ligand efficiency metrics, ADME/tox and repellency screening are within the threshold values; hence, α-pinene, linalool, and myrcene are safe and fit-to-use in the development of a green and novel repellent.

## 1. Introduction

Malaria has been and continues to be one of the world’s leading causes of mortality. Despite the fact that the disease has been eradicated in the United States, it remains a global health threat. Malaria affects an estimated 300 to 500 million people worldwide each year, resulting in 1.5 to 2.7 million fatalities. Sub-Saharan Africa, on the other hand, accounts for about 90% of all malaria cases worldwide [1], followed by the World Health Organization (WHO) South-East Asia Region (3.4%) and Eastern Mediterranean Region (2.1%). Nearly 85% of the worldwide malaria burden was carried by 19 nations in Sub-Saharan Africa and India. Nigeria (25%), the Democratic Republic of the Congo (12%), Uganda (5%), Côte d’Ivoire (4%), Mozambique (4%), and Niger (4%) accounted for more than half of all malaria cases globally [2]. In addition, one to two million people suffering from malaria die from the disease, the majority of whom are children under the age of five and pregnant women [3]. Unfortunately, 90% of malaria deaths occur in Sub-Saharan Africa, where approximately 3000 people die each year. The Nigerian situation is consistent with the global trend, as malaria is the leading cause of death among pregnant women in the country, accounting for one out of every ten deaths [4,5].

Malaria is caused by a species of *Plasmodium* which is vectored by the adult female *Anopheles* mosquito that thrives in hot and humid climates around the world [6]. The *Anopheles gambiae* complex consists of at least seven morphologically similar but six genetically and behaviorally distinct mosquito species from the genus *Anopheles*. The complex contains the most important malaria vectors in Sub-Saharan Africa, especially those that transmit *Plasmodium vivax*, *Plasmodium ovale*, *Plasmodium malariae*, *Plasmodium falciparum*, and *Plasmodium knowlesi* [7]. *A. gambiae* is more than just a pest and a nuisance; it is also responsible for the spread of malaria and other deadly diseases across Africa. 

Quinoline-based drugs were the mainstay of malaria treatment and prevention for decades. Unfortunately, the emergence of drug-resistant Plasmodium species as a result of mutation has rendered conventional therapeutic treatment ineffective [8,9]. WHO, on the other hand, has approved insecticides, fumigation, air shields, ultrasonic rays, pesticide spraying, insecticidal treated nets, and insecticidal-treated clothing as malaria prevention measures [10]. For a variety of reasons, insect repellents such as lotions, coils, mosquito-treated nets, and liquidators have limited efficiency [11,12]. However, consumers have recently become more interested in cheap plant-based commercial repellents, which are frequently considered as “safe” when compared to synthetic repellents; this is not always the case depending on the secondary metabolites present [13]. Plant-based metabolites have been shown to be an effective mosquito control alternative to synthetic insecticides or when used in conjunction with other pesticides as part of an integrated vector control strategy [14].

Several plant extracts have been reported to have mosquitocidal growth regulator activity, as well as insecticidal properties in the elimination of larval or adult mosquitos or as mosquito repellents to protect against mosquito bites [13,15,16]. *Vitex negundo* L. (Verbenaceae) is a well-known medicinal herb [17,18,19,20], which has been linked to a variety of pharmacological properties, including enzyme inhibition [21,22], antifeeding [23], larvicidal [24], and mosquito repellent activities [15]. 

Research on synthetic insect repellents has developed due to breakthroughs in our understanding of mosquito behavior and olfactory receptors [25], but there have been insufficient studies on the safety of plant-based mosquito repellents and their interaction with olfactory receptors. The olfactory system of insects is made up of a number of transmembrane odorant receptor proteins that are expressed in olfactory membrane neurons all throughout their bodies [26]. Odorant binding proteins (OBPs) are potent mosquito biosensors, according to Di Pietrantonio et al. [27], Sankaran et al. [28], and Possas-Abreu et al. [29]. Odorant-binding protein 1 (OBP1) (PDB ID 3N7H) and (OBP:PDB ID 2ERB), odorant-binding protein 7 (OBP7: PDB ID 3R1O), and odorant-binding protein 4 (OBP4: PDB ID 3Q8I) are the key olfactory proteins involved in signals for host recognition process, repellent alarm pheromone, and diphenyl ester-specific binding protein, respectively.

More systematic research is needed to better evaluate plant-based repellents and produce novel solutions that are safe for consumers. This study investigates the major constituents of *V. negundo* essential oil in order to establish repellent efficacy, predict their in-silico toxicity profile, and determine the interactions with *Anopheles* odorant binding proteins using a molecular docking-based technique.

## 2. Materials and Methods

### 2.1. Collection Sites and Identification of V. negundo Leaves

The leaves of *V. negundo* were harvested in September 2020 from six states of the North-Central Geopolitical Zone of Nigeria with the climatic condition and major soil type presented in Table 1. Samples of the leaves were identified at the Department of Medicinal Plant Research and Traditional Medicine, National Institute of Pharmaceutical Research and Development (NIPRD) Idu, Abuja and voucher specimens NIPRD/Hebarium/1101 were deposited.

### 2.2. Leaf Processing and Extraction of Essential Oils

**Figure 1:**
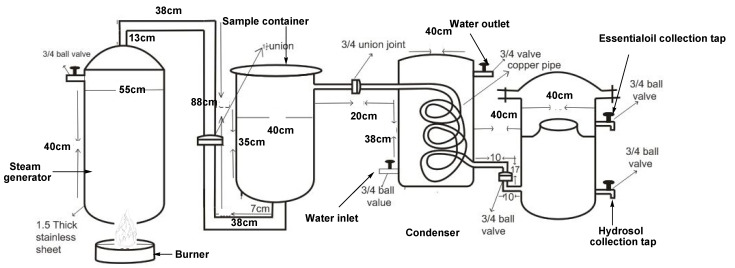
Schematic of the Essential oil Distillation System (EDS).

Collected fresh *V. negundo* leaves were washed with tap water and extracted within 12 h of collection using a 25 kg capacity fabricated Essential oil Distillation System (EDS) based on the steam distillation principle (Figure 1). The EDS steam generator was filled with 50 L of distilled water while the sample container was loaded to capacity and distilled over a period of 45 min. The distillate was recovered and separated in batches using a 2 L separatory funnel into essential oil and aqueous distillate (hydrosol), after which the essential oils were dried over anhydrous Na_2_SO_4_ and stored for further analysis. Finally, the oil yield was calculated relative to the fresh matter and the result presented as the mean ± standard deviation of triplicate extractions. 

### 2.3. GC-MS Profiling of the Essential Oils

The GC-MS analyses of the essential oils were performed with a Varian CP-3800 gas-chromatograph equipped with a HP-5 capillary column (30 mm × 0.25 mm; coating thickness 0.25 μm), carrier gas nitrogen at 1.2 mL/min, and a Varian Saturn 2000 ion trap mass detector. The oven temperature was programmed from 50 to 280 at 3 °C/min. Analytical conditions: injector and transfer line temperatures were 220 and 240 °C, respectively. Volume injected: 0.2 μL of 10% hexane solution, split ratio 1:30. Co-injection of the essential oil with a solution containing a similar series of C8–C22 n-alkanes yielded linear retention indices for all molecules. Retention indices were used to identify the individual components, which were then compared to compounds previously reported in the literature [30,31]. Further, the identification of the compounds was made using data of a computer library (Wiley 275L) connected to the GC-MS, Adams library (https://b-ok.cc/book/3506611/3b1f4f (accessed on 15 October 2021)), the NIST website (https://webbook.nist.gov/chemistry/ (accessed on 15 October 2021)) using RI values from comparable polarity columns, and/or the Mondello library (https://www.sisweb.com/software/wiley-ffnsc.htm (accessed on 15 October 2021)).

### 2.4. Sampling, Rearing, and Identification of Mosquitoes

In the months of March and April 2021, mosquito larvae were collected from selected localities in Kaduna metropolitan located between 10°33′ N of the equator and 07°27′ E of the Greenwich Meridian (Ungwan Gwari; 10°35.71′; 07°27.17′ Ungwan Romi 10°25.19′; 07°25.20′ Kamanzou 10°46.24′; 07°49.41′). The larvae were collected using a 7 cm diameter, 5 cm deep, and 30 cm long handle plastic standard dipper from a 0.12 m × 2.5 m deep temporary pool with grass vegetation. At the breeding sites, larvae identification and morphological classification were carried out. The absence of a siphon, the parallel swimming pattern on the water surface, and the morphology of the combs were used to sort larvae into the Anophelinae and Culicinae subfamilies under a compound microscope, compared to the Culex larva with a long siphon, lighter color, and “hairy” body, as well as the identification key of Gillies and Coetzee [32]. The immature larval stages were carefully transported in vials to the Insectary at the Biological Sciences laboratory, Kaduna State University, Nigeria. The larvae were then placed in an open plastic container 29 cm × 21 cm × 30 cm containing 1 L of ground water and allowed to acclimate for 2 h before being fed finely ground low-fat flour-baked food product [33]. 

**Figure 2:**
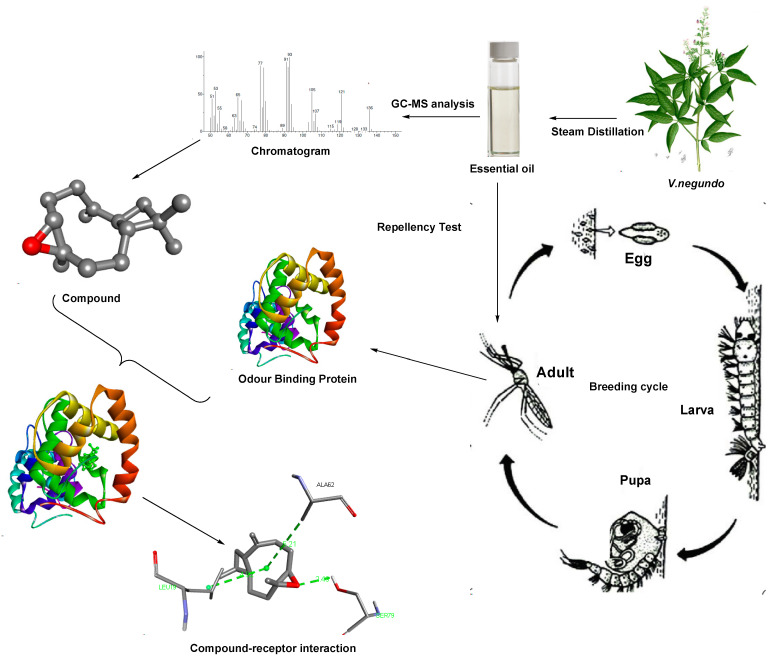
Graphical illustration of repellency and odorant binding protein efficiency using a molecular docking-based technique.

The larvae were batched in separate breeding containers and were reared to adults in separate 30 cm × 30 cm wooden made net chambers for three weeks under controlled optimum conditions of 25 °C, 65% relative humidity, and regulated light/dark (14/10 h) cycle. The emerged adults were identified morphologically using taxonomic characters such as the palps, proboscis, wing venation, and markings or tuffs on legs or abdomen as provided by the dichotomous keys employed by Coetzee and Gillies [34]. This was performed using the simple Olympus light microscope to genera and species level. The adults in the cages were fed a 10% sucrose solution after eclosion from their pupal cases and allowed to rest and mature for 2 to 3 days. Only newly emerged adult females *A. gambiae* were manually aspirated into a 200 mL perforated plastic container and allowed to rest for 1 hr before exposure to the essential oils (Figure 2).

### 2.5. Anopheles Species Authentication: Genomic DNA Extraction and PCR Amplification

The emerged adult mosquitoes belonging to the *A. gambiae* (s.l) complex were subjected to PCR and genomic DNA assays designed for species, molecular form identification, and molecular analysis to fully authenticate the adult *Anopheles* species. The molecular analyses were conducted at the Centre for Biotechnology Research and Training, Ahmadu Bello University, Zaria. Genomic DNA (gDNA) was extracted from 20 Anopheles adult mosquitoes using the Quick-DNA™ Miniprep Plus Kit (D4069) product by ZYMO research company according to the protocol of the manufacturer.

Genomic mosquito DNA (2 μL) was extracted and placed in a 0.2 mL thin-walled Eppendorf tube and 23 μL of the PCR reaction mixture containing species specific primers for *A. gambiae* (GA = 5′-CTGGTTTGGTCGGCACGTTT-3′), *A. Arabiensis* (AR = 5′-AAGTGTCCTTCTCCATCCTA-3′), and a unique primer for all species (UN = 5′-GTGTGCCCC TTCCTCGAT GT-3′) according to the protocols of Scott et al. [35] and Favia et al. [36]. Then, deoxynucleotide triphosphates, magnesium chloride, PCR buffer, distilled water, and recombinant Taq DNA polymerase were all added. Amplification was performed in an initial denaturation step at 94 °C for two minutes, then a 30-cycle denaturation at 94 °C for 30 s, annealing at 49 °C for 30 s and elongation at 68 °C for 5 min in a thermal cycler machine. The PCR amplicons ran on a 1.5% agarose gel electrophoresis gel tank and were viewed under the trans-illuminator UV light for the characteristic *A. gambiae* positive band sizes at 390 bp.

Again, from the samples that showed base pairs of 390 band sizes (*A. gambiae* s.l), seven out of a group of 20 were randomly selected and 1 μL picked and mixed in 24 μL of PCR reaction mixture containing species specific primers for *A. gambiae* s.s and coluzzi. Species identification of *A. gambiae* (s.l.) was performed by PCR according to Favia et al. [36]. The PCR conditions were 10 min at 94 °C as the initial step, followed by 30 cycles (94 °C for 30 s, 53 °C for 30 s, and 68 °C for 30 s). After the last cycle, the products were finally extended for 5 min at 68 °C. Primers used in the PCR were: R5 (5′-GCC AAT CCG AGC TGA TAG CGC-3′), R3 (5′-CGA ATT CTA GGG AGC TCC AG-3′), Mop int (5′-GCC CCT TCC TCG ATG GCA T-3′), and B/S int (5′-ACC AAG ATG GTT CGT TGC-3′). Amplified fragments were analyzed on a 1.5% agarose gel for the characteristic band size of 475 for *A. gambiae* s.s.

### 2.6. Mosquito Behavioral Study Repellency Y-Tube Olfactometer Test

This experiment used an olfactometer with a glass Y-tube of 1.6 cm diameter, 12-cm base, and two 40.62 cm arms at a 45° angle to one another [37]. *V. negundo* essential oil diluted with paraffin oil (analytical purity, Sigma-Aldrich) was added in a gradient sequence of 0.1, 0.25, 0.5, 0.75, and 1.0% *v*/*v* to a 5 g cotton ball, which was then mounted on the cotton ball holder of the test arm. 

The cotton ball holder on the control arm of the glass Y-tube supported the negative control cotton ball (paraffin oil without the essential oil). At a rate of 180 mL/min, fresh air was provided by an electric air pump and filtered on a carbon and silicone base with air flowing into the respective arm of the Y-tube. 

The essential oil was combined with the purified air and went through one arm of the Y-tube whereas purified air with no essential oil went through the opposite arm. Precisely, 100 female *A. gambiae* mosquitoes were discharged into the glass Y-tube, where behavioral responses were monitored and recorded for 1800 s [38]. In addition, to establish the mosquito behavioral activity and the degree of synergism between the pure constituents of the essential oil, selected commercially available pure constituents based on the absorption, distribution, metabolism, excretion, and toxicity (ADME/tox) and docking studies were evaluated. After each investigation, the Y-tubes were air cleaned with a stream of hot air (>60 °C), the cotton ball was removed, and the holder was cleaned. The olfactory test was repeated thrice. The repellent rate was calculated according to the following Equation (1) [39,40]:

(1) $$\%\text{Mosquitoe Repelled}=100-\left(\frac{\text{mean number of mosquitoes selecting essential oil}}{100-\text{mean number of mosquitoes not selecting essential oil}}\right)100$$

The % mosquito repelled of 50% individuals (ED_50_) was estimated using the Probit analysis model in the IBM SPSS v.25 statistical software.

### 2.7. Target Protein Selection and Preparation 

**Figure 3:**
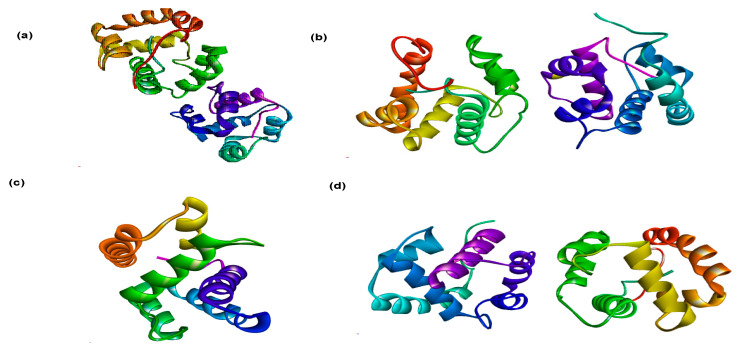
3D structures of *A. gambiae* selected (**a**) OBP 1, (**b**) OBP 7, (**c**) OBP 4, and (**d**) OBP.

The odorant binding proteins (OBPs) were selected as a target based on their application as bio-recognition elements and biosensors for small ligands. The three-dimensional (3D) structures of four *A. gambiae* OBPs; OBP 1 (PDB ID 3N7H), OBP 7 (PDB ID 3R1O), OBP 4 (PDB ID 3Q8I), and OBP (PDB ID 2ERB) were retrieved from the Protein Data-bank (http://www.rcsb.org (accessed on 12 February 2021)) (Figure 3). The crystal structures of the of OBPs were processed by removing existing ligands and water molecules while missing hydrogen atoms were added according to the amino acid protonation state at pH 7.0 utilizing Autodock 4.2 (Molecular Graphics Laboratory, Scripps Research Institute, La Jolla, CA, USA). Thereafter, non-polar hydrogens were merged while polar hydrogens were added to each protein. The process was repeated for each protein and subsequently saved into a dockable format.

### 2.8. Ligands Preparation 

**Figure 4:**
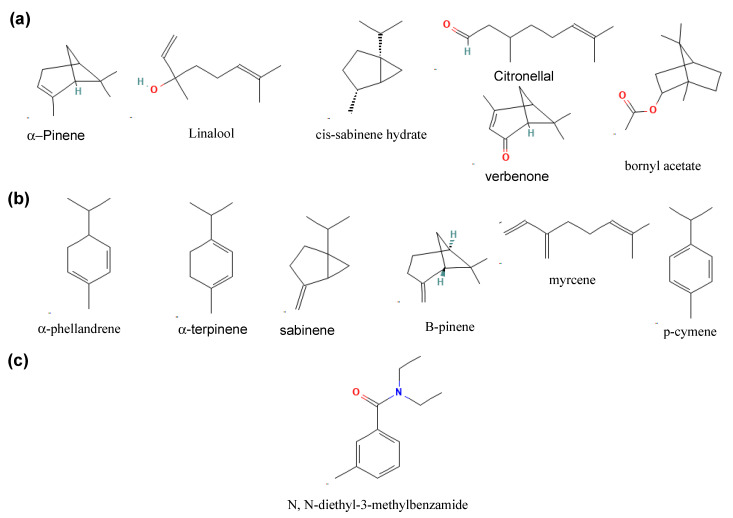
2D structures of the selected ligands (**a**) found in all essential oils irrespective of collection site, (**b**) with percentage composition ≥10%, and (**c**) *N*,*N*-diethyl-3-methylbenzamide (DEET).

The 2D structures of the ligands subjected to docking investigation are presented in Figure 4. In this study, six ligands (α-pinene (PubChem CID 6654), linalool (PubChem CID 6549), cis-sabinene hydrate (PubChem CID 101629835), citronellal (PubChem CID 7794), verbenone (PubChem CID 29025), and bornyl acetate (PubChem CID 6448) identified through the GC-MS analysis in all essential oils irrespective of the collection location were retrieved from the PubChem database (www.pubchem.ncbi.nlm.nih.gov (accessed on 12 February 2021)) in the Structure Data Format (SDF) [41,42,43,44].

An additional six major ligands with percentage composition ≥10% were also selected as potential repellent agents: α-phellandrene (PubChem CID 7460), α-terpinene (PubChem CID 7462), sabinene (PubChem CID 18818), β-pinene (PubChem CID 440967), myrcene (PubChem CID 31253), and p-cymene (PubChem CID 7463). *N*,*N*-diethyl-3-methylbenzamide (DEET) (PubChem CID 4284) was selected as a positive control in this study since it is widely used as a chemical repellent against a variety of insects [45,46] and has strong electrophysiological responses [47]. This is reinforced by DEET’s spatial repellence, acting as a “confusant” and “stimulus” to insects, interfering with odorant detection within the olfactory receptor neurons (ORNs) or odorant receptors (ORs), resulting in avoidance behavior [48].

### 2.9. Molecular Docking Studies

Molecular docking was carried out using PyRx-Python Prescription 0.8 software (Hangzhou, Zhejiang, China). The input file was in the form of PDB code of the receptor or PDB file format and the molecules were in PDB file format. The output file was a docking report. The docked image was viewed by “BIOVIA Discovery Studio Visualizer” software (Waltham, MA, USA) to review the interactions between ligands and proteins, and the length of the interaction along with amino acids. The ligands were imported into PyRx 0.8 through the OpenBabel plug-in tool for each docking phase, with the Universal Force Field (UFF) as the energy minimization parameter, and conjugate gradient descent as the optimization algorithm. Table 1 shows the coordinate of the active sites of the four *A. gambiae* odorant binding proteins as determined by the grid boxes utilized in the docking studies. 

The inhibition constant and ligand efficiency metrics were quantitatively estimated using Equations (2)–(7) [49,50,51,52,53].

(2) $$\mathrm{Inhibition}\;\mathrm{constant}\;\left(k_{i}\right)=exp^{\left(\delta Binding\;Energy\;\left(BE\right)/RT\right)}$$

(3) $$\mathrm{Ligand}\;\mathrm{Efficiency}\;\left(\mathrm{LE}\right)=\frac{-BE}{HA}$$

(4) $$\mathrm{Ligand}\;\mathrm{Efficiency}\;\mathrm{Scaled}\;(LE_{scale})=0.873e^{-0.026\left(HA\right)}-0.064$$

(5) $$\mathrm{Ligand}\;\mathrm{Lipophilic}\;\mathrm{Efficiency}\;\left(LLE\right)=-\left(logk_{i}+logP\right)$$

(6) $$\mathrm{Fit}\;\mathrm{Quality}\;\left(FQ\right)=\frac{LE}{LE_{scale}}$$

(7) $$\mathrm{Ligand}\;\mathrm{Efficiency}\;\mathrm{Lipophilic}\;\mathrm{Price}\left(LELP\right)=\frac{Log\;P}{LE}$$

### 2.10. Absorption, Distribution, Metabolism, Excretion, and Toxicity (ADME/tox) Investigation

The ADME/tox filtering analysis of all selected ligands for docking including the physiochemical were predicted using the ADMETlab 2.0 webserver (https://admetmesh.scbdd.com/service/evaluation/index (accessed on 15 October 2021)). The canonical SMILES of the ligands downloaded from the PubChem Database were used for the calculation of the ADME/tox parameters in default mode.

## 3. Results and Discussion

### 3.1. V. negundo Essential Oil Yields, Climate and Soil Type of the Six States

Table 2 indicates the general soil types, climatic conditions, temperature, and annual rainfall range of the collection sites, as reported in previous literature [54,55,56,57,58,59], and essential oil yield of the six states. Ferralsols and hydromorphic tropical soils were the predominant soil type of Kwara, while the soils of the Plateau are poorly drained sandy clay loam surfaces with clay subsurface. The Niger, Nasarawa, and Benue are dominated by sandy loamy soil types with a clay subsoil as well, except for Kogi soil which is richly sandy and loamy.

The oil yields were generally high for all sampled states; however, *V. negundo* from Niger and Kogi produced the highest and lowest essential oil yields, respectively. Besides, there was no significant difference (*p* > 0.05) in the yields from Nasarawa, Plateau, and Kwara. In accordance with the findings of Tirillini et al., [60], the essential oil yield and composition were influenced by Ca^2+^ and K^+^ concentrations, percentage of organic matter, and temperature. The high yields in Niger, Nasarawa, Benue, Kwara, and Plateau may be attributable to their proximity to a region with a moderately low to low annual rainfall, low temperature, and predominant loamy soil with clay substructure with significant concentrations of K^+^, Na^+^, and Ca^2+^ [61]. However, the significantly high yield obtained in Niger is justifiably due to the low annual rainfall (59.9–274.2 mm) compared to other regions with the same soil substructure (Table 2). On the other hand, the low yield recorded in the Kogi sample may be directly linked to the lack of clay minerals in the soil.

### 3.2. Chemical Composition of V. negundo Essential Oils

Table 3 shows the results of the *V. negundo* essential oils GC-MS analysis collected from six states and the chromatograms are presented as Supplementary Figures S1–S6. The essential oil from the various collection sites showed compositional variation. Niger, Kwara, Benue, Plateau, Kogi, and Nasarawa oil, respectively, contained 16, 18, 30, 24, 15, and 28 identified compounds. Monoterpenes made up the majority of the constituents of the essential oils across all study states with about 74.65–96.23%, followed by about 0.75–16.32% sesquiterpene content. The rest were other compounds of about 3.55–10.88%. Essential oils from Niger, Kwara, and Kogi had more than 90.04% monoterpene while oils from Plateau, Nasarawa, and Benue demonstrated the highest sesquiterpene content (7.63–16.32%). Sesquiterpene content was found to be quite low in Niger and Kwara (0.75–1.23%), with sesquiterpene completely absent in the Kogi sample. The observed compounds are in consonant with the reports of Hebbalkar et al. [17], Huang et al. [62], and Kumar et al. [63].

Major components (≥10%) such as α-phellandrene were found in Niger (34.65%) and Kwara (20.27%), sabinene in Plateau (12.21%) and Nasarawa (11.31%), β-pinene (42.04%) and p-cymene (16.47%) in Kogi, and myrcene (16.78%) in Benue. As a result, the predominant monoterpenes (≥10%) present in *V. negundo* essential oil throughout the six states were α-phellandrene (20.27–34.65%), sabinene (11.31–12.21%), β-pinene (42.04%), p-cymene (16.47%), and myrcene (16.78%). In the chemotaxonomical classification of *V. negundo*, unique compounds such as α-pinene, linalool, cis-sabinene hydrate, citronellal, verbenone, and bornyl acetate found in all samples irrespective of the collection site could be used in the fingerprint of the essential oil. Several factors, including growth conditions, temperature, altitude, soil type, agricultural methods and practices, developmental stage, plant part extracted, and harvesting period, are strong factors that determine the presence and absence of certain terpenes, according to Moghaddam and Mehdizadeh [64]. Few monoterpenes and sesquiterpenes reported by Issa et al. [65] and Khokra et al. [31] were noticeably absent across all six states; however, compounds such as p-cymene [66], α-terpinolene [67], and citronellal [68] with reported potent insecticidal properties were present in the oils.

### 3.3. PCR Confirmation of Anopheles Gambiae s.s

**Figure 5:**
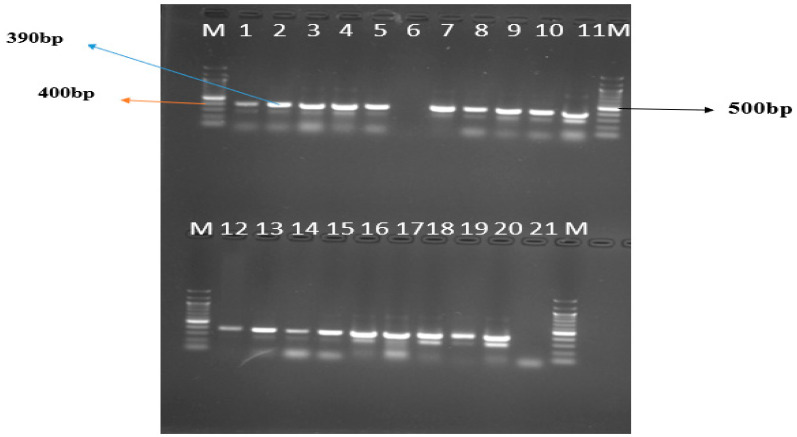
Lane M is the 100 bp marker, Lanes 1–20 are randomly selected *Anopheles* samples. Lane 21 = negative sample. Distinguishing band size: *A. gambiae* s.l *at* 390 bp; *A. arabiensis* 315 bp.

The PCR amplicons viewed under the trans-illuminator UV light showed positive band sizes of 390 bp characteristic for *A. gambiae* while band size of 315 indicates *A. arabiensis* (Figure 5).

**Figure 6:**
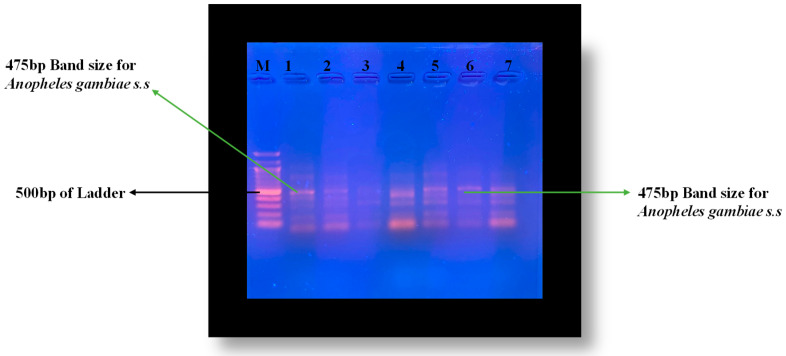
Agarose gel 1.5% for distinguishing *A. gambiae* s.s and *coluzzi* after PCR with primers (R3, R5, B/Sint and MoPint).

After conditioning the PCR, samples 1 to 7 of randomly picked *A. gambiae* s.l showed DNA band sizes of 475 bp, authenticating the species to be *A. gambiae* s.s (Figure 6)

### 3.4. Mosquito Behavioural Study

**Figure 7:**
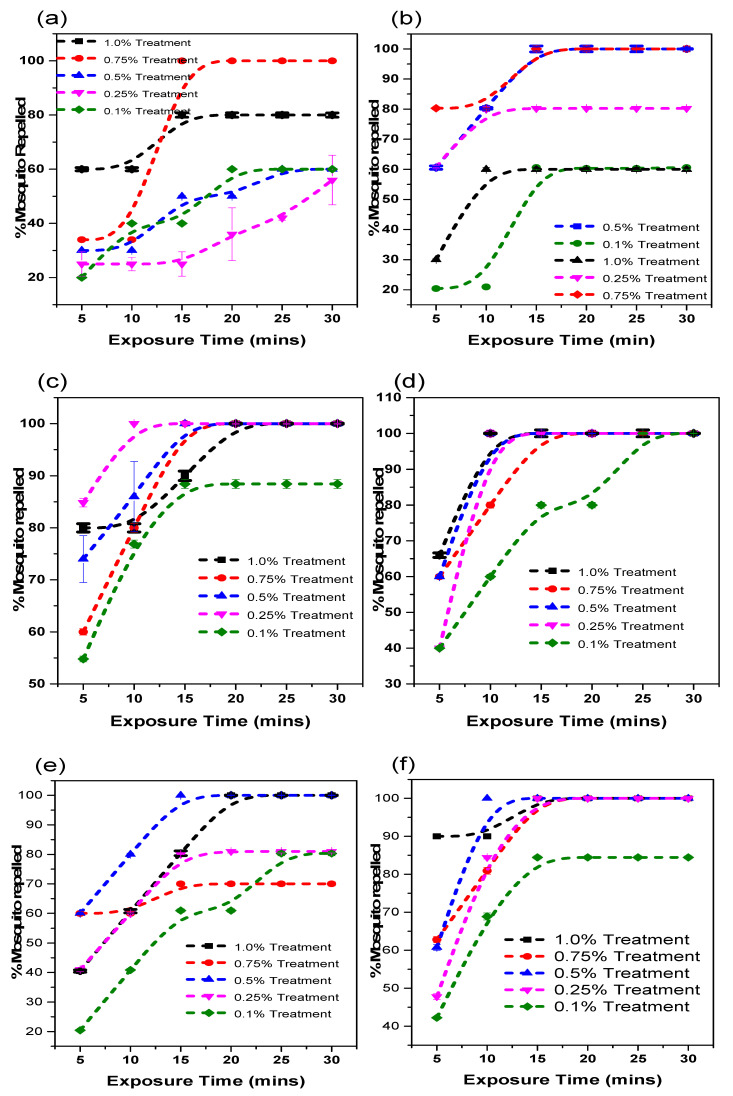
*V. negundo* essential oil obtained from (**a**) Kogi, (**b**) Benue, (**c**) Niger, (**d**) Nasarawa, (**e**) Plateau, and (**f**) Kwara, % mosquito repellency per 5 min at a 30 min exposure periods.

**Figure 8:**
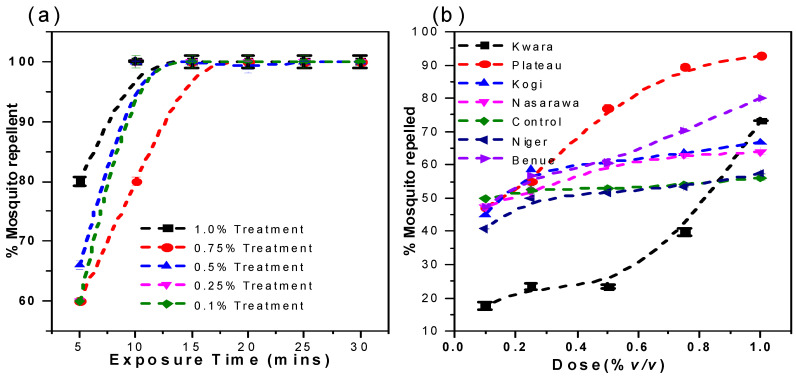
(**a**) % mosquito repellency every 5 min at an exposure time of 30 min to N, N-diethyl-3-methylbenzamide (DEET) and (**b**) Dose response mosquito repellency study of *V. negundo* essential oils obtained from the six states.

The exposure of adult female *A. gambiae* to the essential oil from all six states and *N*,*N*-diethyl-3-methylbenzamide (DEET) for a period of 30 min at doses ranging from 0.1–1% *v*/*v* was investigated and reported in Figure 7 and Figure 8.

There was an increase in the number of mosquitos repelled with time with an optimal % repellency activity attained at approximately 15 min. All the essential oil samples showed a significant increase in the percentage of mosquitoes repelled within the period of investigation with no significant difference in the percentage of mosquitoes repelled (*p* > 0.05) between the essential oils and the *N*,*N*-diethyl-3-methylbenzamide. As the doses increased, the repellency activity increased to a concentration where there are no observable changes in activity. However, the optimal concentration varies from state to state as a result of its compositional variation (Table 3). Essential oils from Niger, Kwara, Plateau and Nasarawa showed optimal repellency at a concentration of 0.5% *v*/*v* while Niger, Benue, and Kogi oil samples showed an optimal effect at 0.75% *v*/*v*. On the contrary, DEET showed no significant difference (*p* < 0.05) in repellency activity as the concentration changed. This inference is in consonance with the studies of Cárdenas-Ortega et al. [69] and Senthil-Nathan [70], which emphasize the slight variation in the repellency activities of samples due to the presence and percentage composition of unique compounds.

### 3.5. Effective Dose of the Essential Oils from the North-Central Geopolitical Zone 

Using the Probit analysis model, the effective dose (ED_50_) that would repel 50% of the mosquito population is presented in Table 4. The ED_50_ of the oils and positive control are in the order of DEET > Kwara > Niger > Plateau and Nasarawa > Benue > Kogi. There is a significant different (*p* < 0.05) in the repellency of the oils from Kwara, Niger, Plateau, and Nasarawa compared to Benue and Kogi. The repellency property of the oils showed a composition–concentration dependent activity, which is not in variance with the result obtained in the mosquito behavioral investigation (Figure 7 and Figure 8). However, DEET showed very potent repellency at ED_50_ of 0.01%*v*/*v* compared to the oils from all states. This observation is due to the variation in the composition.

### 3.6. Validation of Molecular Docking Protocol 

**Figure 9:**
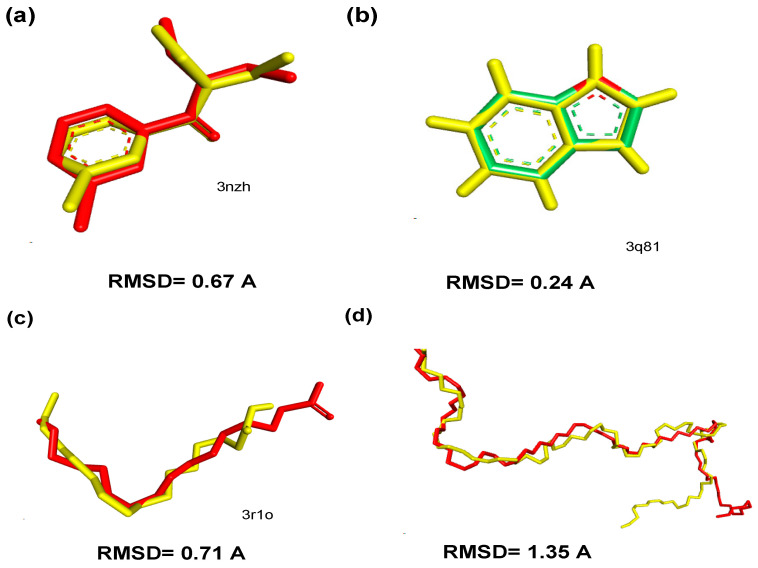
Crystallographic complexes (in red) overlapping with estimated poses (in yellow): (**a**) OBP 1 (PDB 3N7H), (**b**) OBP 4 (PDB 3Q8I), (**c**) OBP 7 (PDB 3R1O), and (**d**) OBP (PDB 2ERB).

According to the literature, a validated protocol must have a RMSD value < 2.0 in the binding mode prediction, when superimposed on the crystallographic pose of the ligand [71,72]. To establish that the conformation of the interaction between co-crystallized ligands and OBPs can be replicated *in silico* to validate our docking method, the co-crystallized ligands were redocked in the protein binding pocket and the root mean square deviation (RMSD) data were used to evaluate the fitness of each redocked pose. Figure 9 illustrates the poses estimated in relation to the deposited PDB complexes, with the RMSD of 0.67 Å, 0.24 Å, 0.71 Å, and 1.35 Å for OBP1, OBP 7, OBP 4, and OBP; respectively.

### 3.7. Molecular Docking 

The binding energies and inhibition constants of the proteins with the selected ligands are reported in Table 5.

All the selected ligands demonstrated multiplicity of binding properties and varying degrees of interaction within the active pockets of the proteins with the exception of OBP 4 which only has affinity for α-pinene, linalool, verbenone, and β-pinene. In Table 5, α-pinene and myrcene (−6.4 kcal/mol), citronellal (−5.5 kcal/mol), linalool (−5.4 kcal/mol), and myrcene (−5.8 kcal/mol) demonstrated the strongest affinities for OBP1, OBP7, OBP4, and OBP, respectively. As a result, myrcene has been identified as an inhibitor of the main olfactory proteins involved in the signals for host recognition processes (OBP1 and OBP), which aid in the collection and transport of hydrophobic odorants into and through the fluid. Repellent alarm pheromone and diphenyl ester-specific binding proteins were inhibited by citronellal (0.1528 mM) and linalool (0.1089 mM), respectively. According to Bohacek et al. [73] and Hughes et al. [74], a molecule with a low Ki value in the micromolar range but less than 10^−4^ mM qualifies as a lead. The estimated Ki values of α-pinene and myrcene, citronellal, linalool, and myrcene against the four targeted OBPs are within the predicted range which qualifies them as a lead mosquito repellent agent. By implication, the strong binding energies and inhibition constants observed for α-pinene and myrcene, citronellal, linalool, and myrcene when compared to other investigated ligands may be suggestive of functional blocking of the olfactory receptor coreceptor, activation of specific odorant receptors (ORs), inhibition of specific ORs responding to attractants, and/or modulation of multiple ORs causing olfactory confusion, according to Tsitoura et al. [46] and Degennaro et al. [75].

### 3.8. Target OBPs Amino Acid–Ligand Interactions 

**Figure 10:**
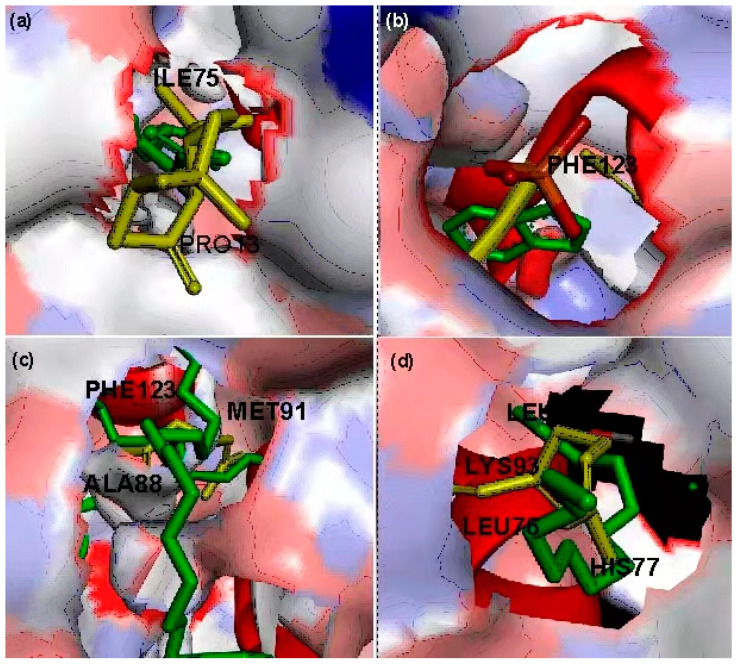
3D images of the active pockets of (**a**) OBP 7, (**b**) OBP 1, (**c**) OBP, and (**d**) OBP 4 showing the native ligand (green) and the experimental ligand (yellow).

Figure 10 illustrates the 3D images of the active pockets of the four selected OBPs while Table 6 reports the list of the active pockets of the four selected OBPs from *A. gambiae*.

The structures of OBP, OBP1, and OBP7 are made up of two monomers, with OBP and OBP1 each having six α-helices and OBP7 having seven α-helices, with the odorant binding pocket placed in the center of a hydrophobic tunnel that runs through the dimeric interface. However, OBP4 is made up of a single monomer. In this investigation, the active pockets of odorant binding proteins were identified by removing the ligands that were previously linked to the receptors before targeting these cavities. The experimental ligands all docked at the same pockets as the native ligands, validating the docking protocol adopted in this study.

**Figure 11:**
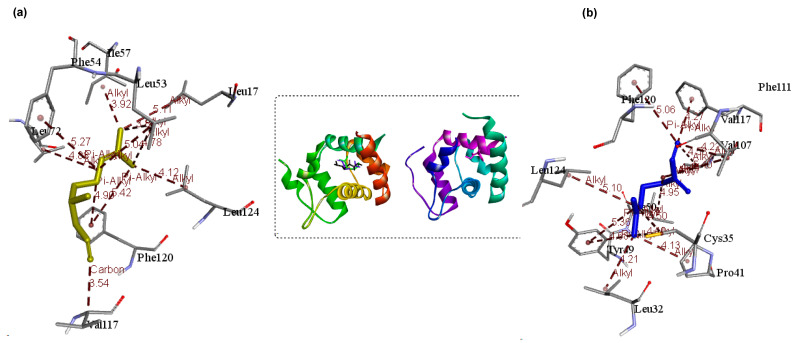
3D interactions showing the selected ligands: (**a**) citronellal, and (**b**) myrcene with the most interaction at the active sites of the OBP 7.

**Figure 12:**
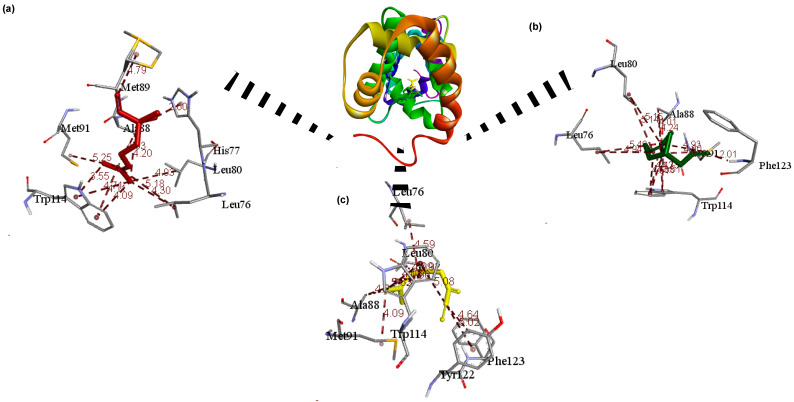
3D interactions showing the selected ligands: (**a**) linalool, (**b**) citronellal, and (**c**) myrcene with the most interaction at the active sites of the OBP.

**Figure 13:**
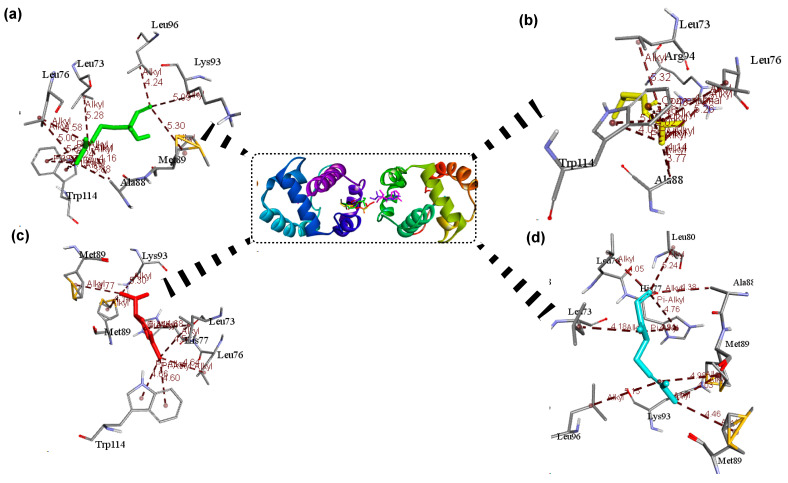
3D interactions showing the selected ligands: (**a**) linalool, (**b**) citronellal, (**c**) α-phellandrene, and (**d**) myrcene with the most interaction at the active sites of the OBP1.

**Figure 14:**
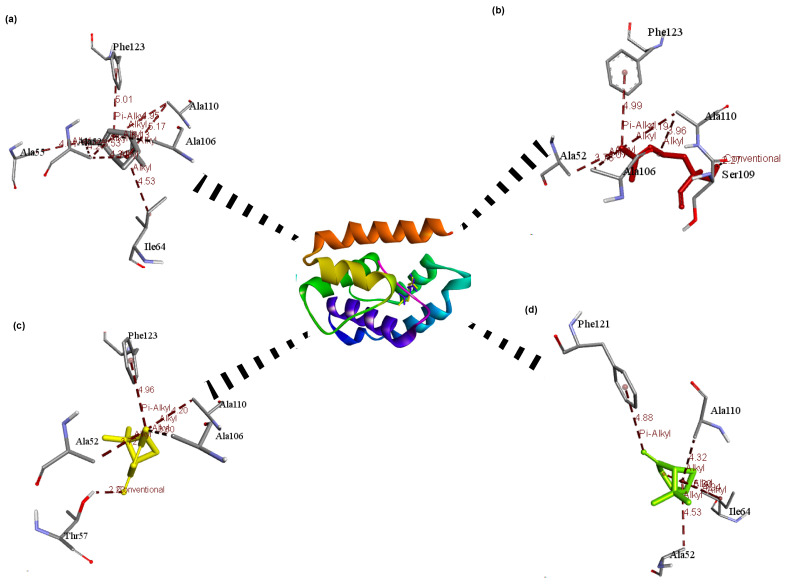
3D interactions showing the selected ligands: (**a**) α-pinene, (**b**) linalool, (**c**) verbenone, and (**d**) β-pinene with the most interaction at the active sites of the OBP4.

Figure 11, Figure 12, Figure 13 and Figure 14 depict the 3D interaction between OBPs and ligands, whereas Table 7 provides the active residues and ligand interaction types. In general, the proteins interact with their ligands primarily through hydrophobic interactions such as π-alkyl and alkyl interactions (Figure 11, Figure 12, Figure 13 and Figure 14). The interaction of the ligands revealed that they bind to at least one receptor in the pocket cavity of the OBPs. The OBPs demonstrated varying affinity for certain ligands as well as variation in the number of residues involved in the interactions. The crystallographic structure of OBP7 was favorably bound to citronellal (−5.5 kcal/mol) and myrcene (−6.2 kcal/mol) through residues in the binding cavity; Leu17 (α-helix 1), Phe120, Leu124 (α-helix 7). and Cys35 (α-helix 2) (Figure 11). Similarly, linalool (−6.2 kcal/mol), citronellal (−6.1 kcal/mol), and myrcene (−5.8 kcal/mol) favorably interacted with OBP through residues Ala88, Met91 (α-helix 5), and Phe123 (α-helix 6) (Figure 12). In the case of OBP1 linalool (−6.2 kcal/mol), citronellal (−6.1 kcal/mol), α-phellandrene, and myrcene (−5.8 kcal/mol) Leu73, Leu76(α-helix 4), Ala88, Met89, Lys93(α-helix 5), Trp114 (α-helix 5) (Figure 13) while OBP4 favorably interacted with α-pinene, linalool, verbenone, and β-pinene through ALA52 (α-helix 3) at a binding energy of ≈ −6.2 kcal/mol (Figure 14).

Interestingly, all major ligand interactions with the OBP, OBP1, OBP4, and OBP7 involve similar residues (Table 7) but differ in the number of interactions as well as distance (Figure 11, Figure 12, Figure 13 and Figure 14). The observed OBP–linalool/citronellal interaction with Ala88 and Met91 involves the 3,7-dimethyl groups of as well as a π-alkyl of the 6-enal interaction on Met 89 at 4.79 Å and on Phe 123 at 2.01 Å; accordingly. OBP-Myrcene complex was formed at the active cavity around Met91 (4.09 Å), Phe123 (4.02 Å), and Ala88 (4.22 Å) (Figure 12). OBP 7 inhibitions were as a result of the following interactions: citronellal: (alkyl, 5.11 Å, Leu17), (pi-alkyl, 4.90 Å, Phe120), (alkyl, 4.20 Å, Leu124), myrcene: (alkyl, 4.13 Å, Csy35), (pi-alkyl, 5.00 Å, Phe120), (alkyl, 5.10 Å, Leu124) (Figure 11). In the case of OBP 4 the inhibitions due to α-pinene (4.11 Å), linalool (3.57 Å), verbenone (3.12 Å), and β-pinene (4.53 Å) were focused at the Ala52 due to alkyl interaction (Figure 14). Consequently, these strong ligand–OBP interactions may result in a functional mutation causing inhibition. 

The mechanisms of interaction between the various ligands differ and will most likely result in a variety of activities ranging from functional blocking of the olfactory receptor coreceptor due to repression of Leu73 in OBP1, inhibition of specific ORs responding to attractants, and/or modulation of multiple Ors causing disorientation, as reported by Murphy et al. [76]. A strong affinity of OBP7 for citronellal and myrcene, according to Sun et al. [77], could create disturbance in the insect’s chemical information decoding potential. These rare interactions of α-pinene, linalool, verbenone, and β-pinene with OBP4 are strongly associated with their spatial orientation of the dialkyl and π-alkyl groups; with the likelihood of blocking the olfactory receptor coreceptor and modulate OBP4, resulting in the susceptibility of *A. gambiae* to these molecules in the repellent.

### 3.9. Efficiency Metrics of Selected Ligands 

Supplementary Tables S1–S4 show the ligand efficiency metrics of the selected ligands, which were calculated using Equations (3)–(7). Ligand Efficiency (LE), Ligand Lipophilic Efficiency (LLE), and Fit Quality (FQ) are expected to have threshold values of 0.3, 3, and 0.8 for a molecule to be classified as a hit quantitatively [78]. During lead discovery, the Ligand Efficiency Lipophilic Price (LELP) is estimated to be between −10 and 10 [79]. The ligand efficiency metrics against the four OBPs are within the criteria, qualifying them as a possible odorant binding protein repellent lead. 

### 3.10. In Silico ADMET Properties of the Ligands against the Odorant Binding Proteins 

#### 3.10.1. ADMET Properties 

The ADMET properties of all selected ligands were carried out to determine the molecules as safe potential OBP inhibitors and the results are presented in Tables S5–S16. Even though cis-sabinene hydrate, citronellal, sabinene, and verbenone failed the human oral bioavailability test (values were 0.7–1.0), this pharmacokinetic parameter is less of a concern regarding skin sensitization and eye irritation for dermally applied products such as repellent lotion or aerosols. In this investigation the empirical decision for skin sensitization and eye irritation tests for linalool, cis-sabinene hydrate, citronellal, sabinene, verbenone, α-terpinene, bornyl acetate, β-pinene, and α-phellandrene are >0.8, which is within the rejection zone because such molecules could induce allergic contact dermatitis, cornea, and conjunctiva tissue damage [80]. Furthermore, citronellal and α-phellandrene have been discovered to be respiratory and human hepatotoxicants, respectively, with high morbidity and mortality potential [81]. The plasma protein binding and blood-brain barrier penetration of cis-sabinene hydrate, α-phellandrene, and α-terpinene were found to be greater than 90%, indicating that these compounds have a low therapeutic index [82]. The metabolic profile of the ligands indicated that they are all either substrates or inhibitors of human cytochrome P450 based on chemical biotransformation reactions [83].

#### 3.10.2. In-silico Environmental Toxicity

To estimate the environmental impact of the essential oil, and the bioconcentration factor (BCF), the concentration of the selected ligands in water in mg/L that causes 50% growth inhibition of Tetrahymena pyriformis after 48 h (IGC_50_), 50% of fathead minnow to die after 96 h (LC_50_), and 50% of Daphnia magna to die after 48 h (LC_50_DM) were evaluated. The result of the analysis is presented in Table 8.

Bioconcentration factors range from 0.553–3.003 L/kg, reflecting the very low potential for the ligands to bioaccumulate in the environment. Citronellal, verbenone (with high human oral bioavailability) (Tables S10 and S12), linalool, and myrcene demonstrated the lowest BCF compared to β-pinene, p-cymene, sabinene, α-pinene, and *cis-sabinene* hydrate (Table 8). The IGC_50_, LC_50_, and LC_50_DM of the selected molecules are 3.080–4.675 (mg/L)/(1000 ∗ MW), 3.547–5.624 (mg/L)/(1000 ∗ MW), and 4.176–5.948 (mg/L)/(1000 ∗ MW), respectively. All studied ligands demonstrated very low ecotoxicological profiles against *Tetrahymena pyriformis* after 48 h, fathead minnow after 96 h, and *Daphnia magna* after 48 h [84]. 

### 3.11. Repellence Study of the Pure Selected Ligands 

**Figure 15:**
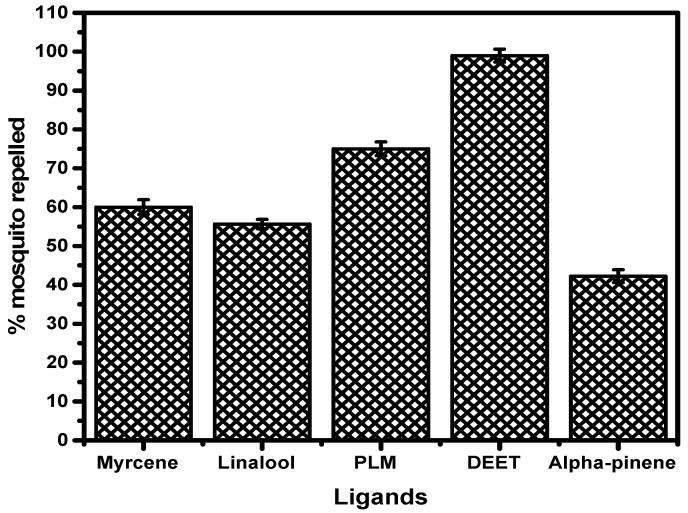
Percentage mosquito repellence test of commercially available main ligands, PLM, and DEET after exposure for 30 min.

Pure α-pinene, linalool, and myrcene could be employed as a safe active ingredient in the development of a new mosquito repellent, according to ADME/tox and docking studies. Exactly 1.0% *v*/*v* of pure α-pinene, linalool, myrcene, equivalent mixture of α-pinene, linalool, myrcene (PLM), and N-diethyl-3-methylbenzamide (DEET) were evaluated for repellent activities and reported after exposure for 30 min (Figure 15). 

The pure linalool and myrcene demonstrated above 50% mosquito repellency activity except for α-pinene. This repellence activity is comparatively lower than the observed activity of the essential oil after 30 min exposure (Figure 7). However, there is a significant difference in the mosquito repellence activity of DEET (100%) compared to the pure ligands (42–60%). Despite the strong interactions of the ligands with the OBPs the observed repellency activity of the essential oil is significantly synergistic in nature. The synergistic activity was validated by comparing the repellence activity of PLM (73%) with DEET (100%) after 30 min of exposure. 

## 4. Conclusions

The essential oils of *V. negundo* from six states in the North Central Geopolitical Zone were found to have composition-dependent mosquito repellent efficacy against *A. gambiae*. In comparison to the other states, the essential oil derived from Niger, Kwara Plateau, and Nasarawa demonstrated substantial repellency with an ED_50_ of 0.14–0.08% *v*/*v*. α-phellandrene, sabinene, β-pinene, p-cymene, and myrcene were the most common terpenes found in the essential oil throughout the six states. Regardless of the collection site, all essential oils contained α-pinene, linalool, cis-sabinene hydrate, citronellal, verbenone, and bornyl acetate. Linalool, α-pinene, verbenone, β-pinene, myrcene, and citronellal had the strongest affinity for OBPs, while -pinene, citronellal, linalool, and myrcene inhibited strongly by creating hydrophobic interactions at the binding pocket. The LE, LLE, FQ, and LELP values were all within the predicted ranges, indicating that the ligands are quantitatively hit and so qualify as a potential odorant binding protein repellent lead. Linalool, cis-sabinene hydrate, citronellal, sabinene, verbenone, α-terpinene, bornyl acetate, α-pinene, and α-phellandrene all had a low ecotoxicological profile, while linalool, cis-sabinene hydrate, citronellal, sabinene, verbenone, and α-terpinene did not. According to ADME/tox and docking results, α-pinene, linalool, and myrcene could be used as safe active components in the development of an environmentally friendly new mosquito repellent. Commercial standards of α-pinene, linalool, and myrcene were themselves active in mosquito repellent assays, and a mixture containing these compounds in equivalent proportions was found to be as significantly active as DEET, suggestive of a synergistic activity itself. Docking showed that these ligands bind to OBPs and may play an important role in blocking the olfactory receptor (ORs) coreceptor and inhibition of specific ORs causing disorientation and confusion in *A. gambiae.*

## Figures and Tables

**Table 1:** Grid box information for the selection of the active pockets of the four odorant binding proteins.

		Centre			Dimension	
Proteins	Center_x	Center_y	Center_z	Size_x	Size_y	Size_z
3N7H	4.552872	15.28167	−12.214	58.59585	78.51029	118.6278
3R1O	4.1755	−10.0047	18.80124	49.47825	50.92539	68.14412
3Q8I	5.995551	1.440093	14.84848	49.47825	49.98114	46.37546
2ERB	2.997585	−0.91365	−39.2475	42.39479	43.98579	64.02445

**Table 2:** *V. negundo* essential oil yields (%), climatic per annum and soil types of the study sites.

States	* Essential Oil Yield (%*w/w*)	Coordinate	Rainfall Range (mm)	Temp. Range (°C)	Major Soil Texture
Kwara	0.13 ± 0.01	8°30 N 5°00′ E	50.8–2413.3	30–35	Ferralsols and hydromorphic tropical soil
Kogi	0.03 ± 0.01	7°30 N 6°42′ E	1200.0–1300.0	15–38	Sandy loamy
Benue	0.29 ± 0.02	7°20 N 8°45′ E	100.0–200.0	21–37	Sandy loamy with sandy clay in the subsoil
Nasarawa	0.15 ± 0.04	8°32 N 8°18′ E	100.1–308.9	14–38	Sandy clay loam and clay loam to clay subsurface.
Plateau	0.18 ± 0.02	9°10 N 9°45′ E	1317.5–1460	12–22	Deep, poorly to very poorly drained sandy clay loam surfaces over clay subsurface
Niger	0.48 ± 0.10	10°00 N 6°00′ E	59.9–274.2	14–38	Loamy sand surface horizons over sandy clay to clay subsurface horizons

* Values are presented as mean of triplicate determination.

**Table 3:** Compositional variation in the essential oils of *V. negundo* from the six study locations.

	Plateau	Nasarawa	Niger	Benue	Kwara	Kogi			
RT	%	%	%	%	%	%	Compounds	RI_Exp_	RI_Lit_
4.711	0.69						α-thuiene	928	924
5.750	40.2	39.83	20.09	27.94	28.76	16.01	α-pinene	934	931
6.789		0.81					camphene	946	943
7.271			1.68	1.28	1.43		sulcatone	965	960
7.800	12.21	11.31	5.99	8.38		4.72	sabinene	983	975
8.255	0.89	1.21			7.94	42.04	β·pinene	988	988
8.289				16.78			myrcene	994	993
8.296			34.65		20.27		α-phellandrene	1005	1002
8.384			8.10	1.44			α·3-carene	1012	1010
8.391					20.36		α-terpinene	1020	1017
8.615			8.57			16.47	p-cymene	1023	1022
8.744	0.69	0.68					β-phellandrene	1025	1025
8.805		0.86		0.65			(E)-β-ocimene	1032	1029
8.812	1.04						y-terpinene	1047	1041
9.060						9.192	trans-sabinene hydrate	1054	1054
9.192	9.11	8.09	4.75	6.72	4.78		Cis-linalool oxide	1088	1086
9.633	0.94	0.92		0.88			trans-linalool oxide	1098	1092
9.708					1.2	0.82	terpinolene	1100	1100
9.776	1.12	1.38	0.77	1.16	0.79	0.67	linalool	1105	1102
9.871	1.31	1.35	0.74	1.03	0.67	0.6	cis-sabinene hydrate	1178	1174
10.47			4.03			2.64	pelargonaldehyde	1193	1186
10.76				1.81			camphor	1318	1316
11.01	1.39	2.01	0.59	2.06	1.79	1.25	citronellal	1395	1389
11.61	2.04	2.42		5.2	2.41		borneol	1414	1409
11.79						0.64	terpinen-4-ol	1425	1417
11.96				1.05			α-terpineol	1445	1437
12.07	1.89	1.00	2.85	1.52	2.84	0.12	verbenone	1456	1452
12.52				0.67			n-decanal	1459	1454
12.61		1.86			1.07	1.72	citronellol	1466	1460
12.61	1.79		1.03	2.87			geraniol	1491	1489
13.01		5.11		3.95			linalyl acetate	1494	1492
13.47	5.65	1.62	2.06	1.94	2.12	1.04	bornyl acetate	1499	1498
13.86		3.47		3.71			4-terpinenyl acetate	1526	1522
14.24	1.19	0.92	1.16		1.2	0.73	α-terplnyl acetate	1552	1548
14.62				0.65			α-cubebene	1561	1561
14.93	3.8	0.67		0.97	0.6		α-ylangene	1574	1574
15.56	2.23	1.23		0.82			α-copaene	1578	1576
15.99	1.09			0.94	0.63		β-bourbonene	1589	1582
16.14	1.96	0.7		0.67			β-elemene	1599	1592
16.56		1.04	0.75	0.77			α-gurjunene	1610	1608
16.68	1.71	1.65		0.64			β-caryophyllene	1637	1638
17.10		1.83		0.68			trans·α·bergamotene	1643	1649
17.19		1.63					α-guaiene	1657	1652
17.20	1.76						α-humulene	1660	1658
17.67		1.59					germacrene D	1871	1880
18.12	1.8	1.92		0.82			β-selinene	1889	1889
18.59	1.97	1.83		0.67			ledene	1891	1890
	1.53	1.06	1.44	1.33	1.14	0.34	Unknown		
Monoterpenes	76.5	74.65	90.04	80.16	94.08	96.232			
Sesquiterpenes	16.32	14.09	0.75	7.63	1.23	0			
Others	5.65	10.2	7.77	10.88	3.55	3.68			

RT: Retention Time (min), RI_Exp_: Experimental retention index, and RI_Lit_: Literature retention index.

**Table 4:** Effective does (%*v*/*v*) of essential oil from the six states and *N*,*N*-diethyl-3-methylbenzamide.

Essential Oil Location	Effective Dose (%*v*/*v*)	R-Square Values
Nasarawa State	0.14	0.8976
Benue State	0.48	0.8995
Kwara State	0.08	0.8254
Plateau State	0.14	0.9778
Niger State	0.11	0.9415
Kogi State	0.87	0.8268
DEET	0.01	0.8942
Petrolatum (Negative control)	-	-

DEET: *N*,*N*-diethyl-3-methylbenzamide.

**Table 5:** Molecular docking results for the interaction between the selected ligands and the odorant binding proteins.

	OBP 1		OBP 7		OBP 4		OBP	
Compounds	BE (kcal/mol)	Ki (mM)	BE (kcal/mol)	Ki (mM)	BE (kcal/mol)	Ki (mM)	BE (kcal/mol)	Ki (mM)
α-pinene	−6.4	0.0201	−6.7	0.0121	−5.8	0.0554	−6.2	0.0282
linalool	−6.9	0.0086	−5.6	0.0777	−5.4	0.1089	−6.2	0.0282
cis-sabinene hydrate	−7.2	0.0052	−6.1	0.0334	-	-	−6.0	0.0395
citronellal	−6.5	0.0169	−5.5	0.1528	-	-	−6.1	0.0334
verbenone	−7.8	0.0019	−7.1	0.0062	−6.1	0.0334	−6.3	0.0238
bornyl acetate	−7.5	0.0031	−7.1	0.0062	-	-	−6.6	0.0052
α-phellandrene	−7.3	0.0044	−7.1	0.0062	-	-	−6.8	0.0102
α-terpinene	−7.3	0.0044	−7.1	0.0062	-	-	−6.8	0.0102
sabinene	−6.7	0.0121	−6.8	0.0102	-	-	−6.9	0.0086
β-pinene	−6.7	0.0121	−6.2	0.0062	−5.9	0.0468	−6.1	0.0334
myrcene	−6.4	0.0201	−6.2	0.0062	-	-	−5.8	0.0554
p-cymene	−7.1	0.0062	−7.1	0.0062	-	-	−6.7	0.0121

BE = Binding energy, Ki = Inhibition constant, and - = No interaction between ligands.

**Table 6:** Active pockets on the four selected odorant binding proteins of *A. gambiae*.

Proteins	Active Pockets
OBP 1	ALA62, LEU73, LEU76, SER79, HIS85, ALA88, MET89, GLY92, LYS93, ARG94, TRP114, PHE123;
OBP 7	PRO13, LEU17, CYS35, ILE75, PHE120, LEU124
OBP 4	THR2, GLN5, HIS29, LYS33, ALA52
OBP	GLU14, ALA18, LEU58, ALA62, SER79, MET84, ALA88, MET89, MET91, ARG94, GLN116, PHE123

**Table 7:** The number and type of bonds for the OBD–ligand complexes.

	Interacting Amino Acids in the Active Pockets			
Ligands	OBP 1	OBP 7	OBP	OBP 4
α-pinene	Leu76, Trp114, Phe123	Phe120, Leu124	Ala88, Met89	Ala52
linalool	Leu73, Leu76, Ala88, Met89, Lys93, Trp114	Cys35, Phe120	Ala88, Met91, Met 89	Ala52
cis-sabinene hydrate	Leu73, Ala88	Phe120	Phe123	Nil
citronellal	Leu73, Leu76, Ala88, Arg94, Trp114	Leu17, Phe120, Leu124	Ala88, Met91, Phe123	Nil
verbenone	Met89, Lys93, Arg94,	Phe120	Phe123	Ala52
bornyl acetate	Trp114, Phe123	Cys35, Phe120	Phe123	Nil
α-phellandrene	Leu73, Leu76, Met89, Lys93, Trp114	Phe120	Ala88	Nil
α-terpinene	Leu73, Met89, Lys93	Phe120	ALA88	Nil
sabinene	Leu73, Ala88, Trp114	Cys35, Phe120	Met89, Met91	Nil
β-pinene	Leu73, Ala88, Met89, Lys93	Cys35	Met91, PHE123	Ala52
myrcene	Leu73, Leu76, Ala88, Met89, Lys93	Cys35, Phe120, Leu124	Ala88, Met91, Phe123	Nil
p-cymene	Leu73, Leu76,Ala88, Trp114	Phe120	Ala88, Met91	Nil

**Table 8:** In-silico environmental toxicity profile of the selected ligands.

Ligands	BCF (L/kg)	IGC_50_ ((mg/L)/(1000 ∗ MW))	LC_50_ ((mg/L)/(1000 ∗ MW))	LC_50_DM ((mg/L)/(1000 ∗ MW))
α-pinene	2.986	4.327	5.287	5.948
linalool	1.347	2.192	3.547	5.056
cis-sabinene hydrate	2.745	3.547	3.657	4.233
citronellal	1.233	3.174	4.168	5.454
verbenone	0.553	3.166	3.989	4.187
bornyl acetate	2.166	3.737	4.334	4.720
α-phellandrene	2.360	3.080	3.674	4.176
α-terpinene	2.246	3.064	4.331	4.538
sabinene	2.874	3.776	4.337	4.400
β-pinene	3.003	4.675	5.624	5.587
myrcene	2.021	4.471	5.331	5.450
p-cymene	2.874	3.776	4.337	4.400

## Data Availability

The data used to support this study are available within the manuscript.

## Supplementary Materials

The following are available online at https://www.mdpi.com/article/10.3390/insects12121061/s1: Figure S1: *V. negundo* essential oil from Benue State, Figure S2: *V. negundo* essential oil from Kogi State, Figure S3: *V. negundo* essential oil from Kwara State, Figure S4: *V. negundo* essential oil from Nasarawa State, Figure S5: *V. negundo* essential oil from Niger State and Figure S6: *V. negundo* essential oil from Plateau State. Table S1: Ligand efficiency metrics of the ligands on interaction with odorant binding protein 1 (PDB ID 3N7H), Table S2: Ligand efficiency metrics of the ligands on interaction with odorant binding protein 7 (PDB ID 3R1O), Table S3: Ligand efficiency metrics of the ligands on interaction with odorant binding protein 4 (PDB ID 3Q8I), Table S4: Ligand efficiency metrics of the ligands on interaction with odorant binding protein (PDB ID 2ERB), Table S5: ADME, Physiochemical, Toxicity, and Environmental Toxicity of Myrcene, Table S6: ADME, Physiochemical, Toxicity, and Environmental Toxicity of α-pinene, Table S7: ADME, Physiochemical, Toxicity, and Environmental Toxicity of β-Pinene, Table S8: ADME, Physiochemical, Toxicity, and Environmental Toxicity of linalool, Table S9: ADME, Physiochemical, Toxicity, and Environmental Toxicity of *cis-sabinene* hydrate, Table S10: ADME, Physiochemical, Toxicity, and Environmental Toxicity of citronellal, Table S11: ADME, Physiochemical, Toxicity, and Environmental Toxicity of α-terpinene, Table S12: ADME, Physiochemical, Toxicity, and Environmental Toxicity of verbenone, Table S13: ADME, Physiochemical, Toxicity, and Environmental Toxicity of bornyl acetate, Table S14: ADME, Physiochemical, Toxicity, and Environmental Toxicity of α-phellandrene, Table S15: ADME, Physiochemical, Toxicity, and Environmental Toxicity of sabinene and Table S16: ADME, Physiochemical, Toxicity, and Environmental Toxicity of p-cymene
