# Supplementary material for: Vitex negundo L. Essential Oil: Odorant Binding Protein Efficiency Using Molecular Docking Approach and Studies of the Mosquito Repellent
Source: Insects. 2021 Nov 26;12(12):1061. doi: 10.3390/insects12121061 (PMC8703855; doi:10.3390/insects12121061)
Supplement: Supplementary file 1 [file insects-12-01061-s001.zip › insects-1461785-supplementary tables.pdf]

Table S1: Ligand efficiency metrics of the ligands on interaction with odorant binding protein 1 (PDB ID **3N7H**)

	**3N7H**				
**Compounds**	**LE**	**LE$_{SCALE}$**	**FQ**	**LLE**	**LELP**
α-pinene	-640	0.6092	-1050.69	-1.2565	-0.0054
linalool	-628	0.5919	-1059.85	-2.4034	-0.0043
cis-sabinene hydrate	-720	0.6092	-1182.02	-1.8735	-0.0048
citronellal	-591	0.5919	-998.41	-1.8299	-0.005
verbenone	-710	0.5919	-1198.09	-3.5838	-0.0031
bornyl acetate	-536	0.5427	-987.24	-2.5037	-0.0056
α-phellandrene	-730	0.6092	-1198.44	-2.3869	-0.0041
α-terpinene	-730	0.6092	-1198.44	-2.0569	-0.0046
sabinene	-670	0.6092	-1099.94	-1.6666	-0.0049
β-pinene	-670	0.6092	-1099.94	-1.4966	-0.0052
rnvrcene	-640	0.6092	-1050.69	-1.2665	-0.0054
p-cymene	-710	0.6092	-1165.61	-1.7102	-0.005

LE: Ligand Efficiency, FQ: Fit Quality, LLE: Ligand Lipophilic Efficiency, LELP: Ligand-efficiency-dependent lipophilicity.

Table S2: Ligand efficiency metrics of the ligands on interaction with odorant binding protein 7 (PDB ID **3R1O**)

	**3R1O**				
**Compounds**	**LE**	**LE$_{SCALE}$**	**FQ**	**LLE**	**LELP**
α-pinene	-670	0.6092	-1099.94	-1.4766	-0.0052
linalool	-510	0.5919	-860.17	-1.4494	-0.0053
cis-sabinene hydrate	-610	0.6092	-1001.44	-1.0663	-0.0056
citronellal	-500	0.5919	-844.81	-1.0961	-0.0059
verbenone	-646	0.5919	-1090.57	-3.0702	-0.0034
bornyl acetate	-508	0.5427	-934.59	-2.2102	-0.006
α-phellandrene	-710	0.6092	-1165.61	-2.2402	-0.0042
α-terpinene	-710	0.6092	-1165.61	-1.9102	-0.0047
sabinene	-680	0.6092	-1116.35	-1.74	-0.0048
β-pinene	-620	0.6092	-1017.85	-1.1297	-0.0056
rnvrcene	-620	0.6092	-1017.85	-1.1197	-0.0056
p-cymene	-710	0.6092	-1165.61	-1.7102	-0.005

LE: Ligand Efficiency, FQ: Fit Quality, LLE: Ligand Lipophilic Efficiency, LELP: Ligand-efficiency-dependent lipophilicity.

Table S3: Ligand efficiency metrics of the ligands on interaction with odorant binding protein 4 (PDB ID **3Q8I**)

			**3Q8I**		
**Compounds**	**LE**	**LEscale**	**FQ**	**LLE**	**LELP**
α-pinene	-580	0.6092	-952.19	-0.8162	-0.006
linalool	-491	0.5919	-829.45	-1.3027	-0.0055
cis-sabinene hydrate	Nil	Nil	Nil	Nil	Nil
citronellal	Nil	Nil	Nil	Nil	Nil
verbenone	-555	0.5919	-936.97	-2.3363	-0.0039
bornyl acetate	Nil	Nil	Nil	Nil	Nil
α-phellandrene	Nil	Nil	Nil	Nil	Nil
α-terpinene	Nil	Nil	Nil	Nil	Nil
sabinene	Nil	Nil	Nil	Nil	Nil
β-pinene	-590	0.6092	-968.6	-0.9096	-0.0058
rnvrcene	Nil	Nil	Nil	Nil	Nil
p-cymene	Nil	Nil	Nil	Nil	Nil

LE: Ligand Efficiency, FQ: Fit Quality, LLE: Ligand Lipophilic Efficiency, LELP: Ligand-efficiency-dependent lipophilicity.

Table S4: Ligand efficiency metrics of the ligands on interaction with odorant binding protein (PDB ID **2ERB**)

			**2ERB**		
**Compounds**	**LE**	**LEscale**	**FQ**	**LLE**	**LELP**
α-pinene	-620	0.6091	-1017.85	-1.1097	-0.0055
linalool	-620	0.6091	-1017.85	-1.1097	-0.0055
cis-sabinene hydrate	-600	0.6091	-985.01	-1.7429	-0.0044
citronellal	-555	0.5919	-936.97	-1.5363	-0.0053
verbenone	-573	0.5919	-967.69	-2.4831	-0.0037
bornyl acetate	-471	0.5426	-868.77	-1.8432	-0.0066
α-phellandrene	-680	0.6091	-1116.35	-2.0199	-0.0044
α-terpinene	-680	0.6091	-1116.35	-1.8979	-0.0045
sabinene	-690	0.6091	-1132.77	-1.8133	-0.0047
β-pinene	-610	0.6091	-1001.43	-1.0563	-0.0056
rnyrcene	-600	0.6091	-985.01	-0.0819	-0.0072
p-cymene	-670	0.6091	-1099.93	-1.4166	-0.0052

LE: Ligand Efficiency, FQ: Fit Quality, LLE: Ligand Lipophilic Efficiency, LELP: Ligand-efficiency-dependent lipophilicity.

Table S5: ADME, Physiochemical, Toxicity, and Environmental Toxicity of Myrcene

	**Adsorption**						
	**Pgp-inh**	**Pgp-sub**	**HIA**	**F(20%)**	**F(30%)**	**Caco-2**	**MDCK**
	0.02	0.001	0.003	0.008	0.011	-4.402	2.23E-05
	**Distribution**				**Metabolism**		
	**BBB**	**5**	**VDss**	**Fu**	**CYP1A2-inh**	**CYP1A2-sub**	**CYP2C19-inh**
	0.852	89.87%	3.235	6.43%	0.785	0.243	0.176
**ADME**	**Metabolism**						
	**CYP2C19-sub**	**CYP2C9-inh**	**CYP2C9-sub**	**CYP2D6-inh**	**CYP2D6-sub**	**CYP3A4-inh**	**CYP3A4-sub**
	0.803	0.073	0.719	0.015	0.519	0.032	0.261
	**Excretion**						
	**CL**	**T0.5**					
	13.108	0.453					
**Physiochemical**	**MW**	**Vol**	**Dense**	**nHA**	**nHD**	**TPSA**	**nRot**
	136.13	173.607	0.784	0	0	0	4
	**nRing**	**MaxRing**	**nHet**	**fChar**	**nRig**	**Flex**	**nStereo**
	0	0	0	0	3	1.333	0
	**LogS**	**LogD**	**LogP**				
	-4.177	3.444	4.321				
**Medicinal Chemistry**	**QED**	**Synth**	**Fsp3**	**MCE-18**	**Natural Product-likeness**	**Alarm NMR**	**BMS**
	0.41	3.081	0.4	0	3.261	0	0
	**Chelating**	**PAINS**	**Lipinski**	**Pfizer**	**GSK**	**Golden Triangle**	
	0	0	Accepted	Rejected	Rejected	Rejected	

	**NR-AR**	**NR-AR-LBD**	**NR-AhR**	**NR-Aromatase**	**NR-ER**	**NR-ER-LBD**	**NR-PPAR-gamma**
**TOX 21 Pathway**	0.012	0.013	0.008	0.024	0.082	0.415	0.112
	**SR-ARE**	**SR-ATAD5**	**SR-HSE**	**SR-MMP**	**SR-p53**		
	0.763	0.01	0.921	0.032	0.093		
**Toxicity**	**hERG**	**H-HT**	**DILI**	**Ames**	**ROA**	**FDAMDD**	**SkinSen**
	0.009	0.61	0.51	0.025	0.022	0.157	0.94
	**Carcinogenicity**	**EC**	**EI**	**Respiratory**			
	0.802	0.925	0.986	0.935			
**Toxicophores**	**Toxicophores**	**Acute Aquatic Toxicity**	**LD50 oral**	**Non-Genotoxic Carcinogenicity**	**Skin Sensitization**	**SureChEMBL**	**Genotoxic Carcinogenicity_ Mutagenicity**
	2	1	0	0	0	1	0
	**Non-Biodegradable**						
	0						
**Environmental Toxicity**	**BCF**	**IGC_{50}**	**LC_{50}**	**$LC_{50}DM$**			
	2.021	4.471	5.331	5.45			

Table S6: ADME, Physiochemical, Toxicity, and Environmental Toxicity of α-pinene

α-pinene	ADME	**Adsorption**						
		**Pgp-inh**	**Pgp-sub**	**HIA**	**F(20%)**	**F(30%)**	**Caco-2**	**MDCK**
		0	0	0.004	0.102	0.102	-4.303	1.83E-05
		**Distribution**				**Metabolism**		
		**BBB**	**5**	**VDss**	**Fu**	**CYP1A2-inh**	**CYP1A2-sub**	**CYP2C19-inh**
		0.896	86.34%	1.73	12.59%	0.469	0.368	0.267
		**Metabolism**						

	**CYP2C19-sub**	**CYP2C9-inh**	**CYP2C9-sub**	**CYP2D6-inh**	**CYP2D6-sub**	**CYP3A4-inh**	**CYP3A4-sub**
	0.867	0.312	0.846	0.012	0.786	0.045	0.263
	**Excretion**						
	**CL**	**T0.5**					
	15.022	0.114					
**Physiochemical**	**MW**	**Vol**	**Dense**	**nHA**	**nHD**	**TPSA**	**nRot**
	136.13	161.767	0.842	0	0	0	0
	**nRing**	**MaxRing**	**nHet**	**fChar**	**nRig**	**Flex**	**nStereo**
	3	0	0	0	8	0	2
	**LogS**	**LogD**	**LogP**				
	-4.662	3.746	4.125				
**Medicinal Chemistry**	**QED**	**Synth**	**Fsp3**	**MCE-18**	**Natural Product-likeness**	**Alarm_NMR**	**BMS**
	0.449	4.273	0.8	30.667	2.714	0	0
	**Chelating**	**PAINS**	**Lipinski**	**Pfizer**	**GSK**	**GoldenTriangle**	
	0	0	Accepted	Rejected	Rejected	Rejected	
**TOX 21 Pathway**	**NR-AR**	**NR-AR-LBD**	**NR-AhR**	**NR-Aromatase**	**NR-ER**	**NR-ER-LBD**	**NR-PPAR-gamma**
	0.006	0.003	0.006	0.005	0.162	0.533	0.005
	**SR-ARE**	**SR-ATAD5**	**SR-HSE**	**SR-MMP**	**SR-p53**		
	0.015	0.003	0.032	0.082	0.002		
**Toxicity**	**hERG**	**H-HT**	**DILI**	**Ames**	**ROA**	**FDAMDD**	**SkinSen**
	0.006	0.196	0.023	0.002	0.021	0.42	0.158
	**Carcinogenicity**	**EC**	**EI**	**Respiratory**			

	0.056	0.955	0.985	0.825			
**Toxicophores**	**Toxicophores**	**Acute_Aquatic_Toxicity**	**LD50_oral**	**NonGenotoxic_Carcinogenicity**	**Skin_Sensitization**	**SureChEMBL**	**Genotoxic_Carcinogenicity_Mutagenicity**
	0	1	0	0	0	0	0
	**NonBiodegradable**						
	0						
**Environmental Toxicity**	**BCF**	**IGC50**	**LC50**	**LC50DM**			
	2.986	4.327	5.287	5.948			

Table S7: ADME, Physiochemical, Toxicity, and Environmental Toxicity of β-Pinene

	**Adsorption**						
	**Pgp-inh**	**Pgp-sub**	**HIA**	**F(20%)**	**F(30%)**	**Caco-2**	**MDCK**
	0.001	0	0.003	0.041	0.004	-4.46	2.05E-05
	**Distribution**				**Metabolism**		
	**BBB**	**5**	**VDss**	**Fu**	**CYP1A2-inh**	**CYP1A2-sub**	**CYP2C19-inh**
	0.986	64.33%	1.091	25.36%	0.296	0.352	0.163
ADME	**Metabolism**						
	**CYP2C19-sub**	**CYP2C9-inh**	**CYP2C9-sub**	**CYP2D6-inh**	**CYP2D6-sub**	**CYP3A4-inh**	**CYP3A4-sub**
	0.825	0.321	0.794	0.009	0.859	0.026	0.252
	**Excretion**						
	**CL**	**T0.5**					
	10.097	0.107					
**Physiochemical**	**MW**	**Vol**	**Dense**	**nHA**	**nHD**	**TPSA**	**nRot**
	136.13	161.767	0.842	0	0	0	0

	**nRing**	**MaxRing**	**nHet**	**fChar**	**nRig**	**Flex**	**nStereo**
	3	0	0	0	9	0	2
	**LogS**	**LogD**	**LogP**				
	-4.389	3.668	3.625				
**Medicinal Chemistry**	**QED**	**Synth**	**Fsp3**	**MCE-18**	**Natural Product-likeness**	**Alarm NMR**	**BMS**
	0.449	4.385	0.8	32	2.973	0	0
	**Chelating**	**PAINS**	**Lipinski**	**Pfizer**	**GSK**	**Golden Triangle**	
	0	0	Accepted	Rejected	Accepted	Rejected	
**TOX 21 Pathway**	**NR-AR**	**NR-AR-LBD**	**NR-AhR**	**NR-Aromatase**	**NR-ER**	**NR-ER-LBD**	**NR-PPAR-gamma**
	0.008	0.003	0.006	0.005	0.199	0.548	0.006
	**SR-ARE**	**SR-ATAD5**	**SR-HSE**	**SR-MMP**	**SR-p53**		
	0.017	0.003	0.017	0.02	0.003		
**Toxicity**	**hERG**	**H-HT**	**DILI**	**Ames**	**ROA**	**FDAMDD**	**SkinSen**
	0.005	0.109	0.051	0.005	0.028	0.714	0.068
	**Carcinogenicity**	**EC**	**EI**	**Respiratory**			
	0.042	0.838	0.982	0.933			
**Toxicophores**	**Toxicophores**	**Acute Aquatic Toxicity**	**LD50 oral**	**Non-Genotoxic Carcinogenicity**	**Skin Sensitization**	**SureChEMBL**	**Genotoxic Carcinogenicity Mutagenicity**
	0	1	0	0	0	0	0

	**Non Biodegradable**			
	0			
**Environmental Toxicity**	**BCF**	**IGC50**	**LC50**	**LC50DM**
	3.003	4.675	5.624	5.587

Table S8: ADME, Physiochemical, Toxicity, and Environmental Toxicity of linalool

	**Adsorption**						
ADME	**Pgp-inh**	**Pgp-sub**	**HIA**	**F(20%)**	**F(30%)**	**Caco-2**	**MDCK**
	0.024	0.002	0.004	0.133	0.019	-4.375	2.27E-05
	**Distribution**				**Metabolism**		
	**BBB**	**5**	**VDss**	**Fu**	**CYP1A2-inh**	**CYP1A2-sub**	**CYP2C19-inh**
	0.953	85.37%	1.509	18.67%	0.163	0.281	0.242
	**Metabolism**						
	**CYP2C19-sub**	**CYP2C9-inh**	**CYP2C9-sub**	**CYP2D6-inh**	**CYP2D6-sub**	**CYP3A4-inh**	**CYP3A4-sub**
	0.832	0.042	0.768	0.05	0.112	0.3	0.285
	**Excretion**						
	**CL**	**T0.5**					
	9.738	0.493					
**Physiochemical**	**MW**	**Vol**	**Dense**	**nHA**	**nHD**	**TPSA**	**nRot**
	154.14	185.034	0.833	1	1	20.23	4
	**nRing**	**MaxRing**	**nHet**	**fChar**	**nRig**	**Flex**	**nStereo**
	0	0	1	0	2	2	1
	**LogS**	**LogD**	**LogP**				
	-2.372	2.426	2.978				

**Medicinal Chemistry**	**QED**	**Synth**	**Fsp3**	**MCE-18**	**Natural Product-likeness**	**Alarm NMR**	**BMS**
	1	1	0	0	0	0	0
	**Chelating**	**PAINS**	**Lipinski**	**Pfizer**	**GSK**	**Golden Triangle**	
	0	0	Accepted	Accepted	Accepted	Rejected	
**TOX 21 Pathway**	**NR-AR**	**NR-AR-LBD**	**NR-AhR**	**NR-Aromatase**	**NR-ER**	**NR-ER-LBD**	**NR-PPAR-gamma**
	0.006	0.003	0.005	0.01	0.112	0.045	0.003
	**SR-ARE**	**SR-ATAD5**	**SR-HSE**	**SR-MMP**	**SR-p53**		
	0.034	0.005	0.272	0.315	0.007		
**Toxicity**	**hERG**	**H-HT**	**DILI**	**Ames**	**ROA**	**FDAMDD**	**SkinSen**
	0.019	0.338	0.022	0.006	0.02	0.019	0.631
	**Carcinogenicity**	**EC**	**EI**	**Respiratory**			
	0.236	0.607	0.988	0.039			
**Toxicophores**	**Toxicophores**	**Acute Aquatic Toxicity**	**LD50_oral**	**Non-Genotoxic Carcinogenicity**	**Skin Sensitization**	**SureChEMBL**	**Genotoxic Carcinogenicity Mutagenicity**
	1	1	0	0	0	0	0
	**Non-Biodegradable**						
	0						
**Environmental Toxicity**	**BCF**	**IGC50**	**LC50**	**LC50DM**			
	1.347	2.192	3.547	5.056			

Table S9: ADME, Physiochemical, Toxicity, and Environmental Toxicity of *cis-sabinene* hydrate

				**Adsorption**			
	**Pgp-inh**	**Pgp-sub**	**HIA**	**F(20%)**	**F(30%)**	**Caco-2**	**MDCK**
	0	0.001	0.005	0.062	0.844	-4.362	2.11E-05
		**Distribution**			**Metabolism**		
	**BBB**	**5**	**VDss**	**Fu**	**CYP1A2-inh**	**CYP1A2-sub**	**CYP2C19-inh**
	0.77	93.18%	1.767	8.76%	0.513	0.722	0.246
**ADME**							
	**CYP2C19-sub**	**CYP2C9-inh**	**CYP2C9-sub**	**CYP2D6-inh**	**CYP2D6-sub**	**CYP3A4-inh**	**CYP3A4-sub**
	0.942	0.285	0.427	0.035	0.548	0.205	0.298
	**Excretion**						
	**CL**	**T0.5**					
	14.938	0.22					
	**MW**	**Vol**	**Dense**	**nHA**	**nHD**	**TPSA**	**nRot**
	138.14	164.403	0.84	0	0	0	1
**Physiochemical**	**nRing**	**MaxRing**	**nHet**	**fChar**	**nRig**	**Flex**	**nStereo**
	2	6	0	0	7	0.143	3
	**LogS**	**LogD**	**LogP**				
	-4.293	4.017	3.751				
	**QED**	**Synth**	**Fsp3**	**MCE-18**	**Natural Product-likeness**	**Alarm NMR**	**BMS**
**Medicinal Chemistry**	0.522	4.116	1	31.2	2.87	0	0
	**Chelating**	**PAINS**	**Lipinski**	**Pfizer**	**GSK**	**Golden Triangle**	

	0	0	Accepted	Rejected	Accepted	Rejected	
TOX 21 Pathway	**NR-AR**	**NR-AR-LBD**	**NR-AhR**	**NR-Aromatase**	**NR-ER**	**NR-ER-LBD**	**NR-PPAR-gamma**
	0.015	0.002	0.003	0.005	0.24	0.413	0.002
	**SR-ARE**	**SR-ATAD5**	**SR-HSE**	**SR-MMP**	**SR-p53**		
	0.018	0.003	0.084	0.036	0.003		
Toxicity	**hERG**	**H-HT**	**DILI**	**Ames**	**ROA**	**FDAMDD**	**SkinSen**
	0.019	0.117	0.323	0.024	0.056	0.037	0.285
	**Carcinogenicity**	**EC**	**EI**	**Respiratory**			
	0.088	0.78	0.975	0.254			
Toxicophores	**Toxicophores**	**Acute Aquatic Toxicity**	**LD50 oral**	**Non-Genotoxic Carcinogenicity**	**Skin Sensitization**	**SureChEMBL**	**Genotoxic Carcinogenicity Mutagenicity**
	0	1	0	0	0	0	0
	**NonBiodegradable**						
	0						
Environmental Toxicity	**BCF**	**IGC50**	**LC50**	**LC50DM**			
	2.745	3.547	3.657	4.233			

Table S10: ADME, Physiochemical, Toxicity, and Environmental Toxicity of citronellal

				**Adsorption**			
ADME	**Pgp-inh**	**Pgp-sub**	**HIA**	**F(20%)**	**F(30%)**	**Caco-2**	**MDCK**
	0.023	0.002	0.006	0.815	0.108	-4.407	2.34E-05
		**Distribution**			**Metabolism**		

	**BBB**	**5**	**VDss**	**Fu**	**CYP1A2-inh**	**CYP1A2-sub**	**CYP2C19-inh**
	0.99	68.87%	3.407	18.17%	0.578	0.406	0.116
	**CYP2C19-sub**	**CYP2C9-inh**	**CYP2C9-sub**	**CYP2D6-inh**	**CYP2D6-sub**	**CYP3A4-inh**	**CYP3A4-sub**
	0.669	0.037	0.728	0.049	0.269	0.033	0.188
	**Excretion**						
	**CL**	**T0.5**					
	13.045	0.418					
**Physiochemical**	**MW**	**Vol**	**Dense**	**nHA**	**nHD**	**TPSA**	**nRot**
	154.14	185.034	0.833	1	0	17.07	5
	**nRing**	**MaxRing**	**nHet**	**fChar**	**nRig**	**Flex**	**nStereo**
	0	0	1	0	2	2.5	1
	**LogS**	**LogD**	**LogP**				
	-2.819	2.945	3.195				
**Medicinal Chemistry**	**QED**	**Synth**	**Fsp3**	**MCE-18**	**Natural Product-likeness**	**Alarm_NMR**	**BMS**
	0.439	3.231	0.7	2	2.567	1	1
	**Chelating**	**PAINS**	**Lipinski**	**Pfizer**	**GSK**	**GoldenTriangle**	
	0	0	Accepted	Rejected	Accepted	Rejected	
**TOX 21 Pathway**	**NR-AR**	**NR-AR-LBD**	**NR-AhR**	**NR-Aromatase**	**NR-ER**	**NR-ER-LBD**	**NR-PPAR-gamma**
	0.005	0.004	0.01	0.007	0.14	0.19	0.004

	**SR-ARE**	**SR-ATAD5**	**SR-HSE**	**SR-MMP**	**SR-p53**		
	0.022	0.005	0.645	0.032	0.076		
**Toxicity**	**hERG**	**H-HT**	**DILI**	**Ames**	**ROA**	**FDAMDD**	**SkinSen**
	0.012	0.508	0.03	0.024	0.011	0.021	0.961
	**Carcinogenicity**	**EC**	**EI**	**Respiratory**			
	0.491	0.976	0.987	0.859			
**Toxicophores**	**Toxicophores**	**Acute-Aquatic Toxicity**	**LD50 oral**	**Non-Genotoxic Carcinogenicity**	**Skin Sensitization**	**SureChEMBL**	**Genotoxic Carcinogenicity Mutagenicity**
	1	0	0	0	2	1	0
	**Non-Biodegradable**						
	1						
**Environmental Toxicity**	**BCF**	**IGC50**	**LC50**	**LC50DM**			
	1.233	3.174	4.168	5.454			

Table S11: ADME, Physiochemical, Toxicity, and Environmental Toxicity of α-terpinene

				**Adsorption**			
ADME	**Pgp-inh**	**Pgp-sub**	**HIA**	**F(20%)**	**F(30%)**	**Caco-2**	**MDCK**
	0.474	0.017	0.004	0.019	0.103	-4.475	2.02E-05
		**Distribution**			**Metabolism**		
	**BBB**	**5**	**VDss**	**Fu**	**CYP1A2-inh**	**CYP1A2-sub**	**CYP2C19-inh**
	0.451	93.25%	2.857	9.54%	0.549	0.873	0.14

	**CYP2C19-sub**	**CYP2C9-inh**	**CYP2C9-sub**	**CYP2D6-inh**	**CYP2D6-sub**	**CYP3A4-inh**	**CYP3A4-sub**
	0.948	0.12	0.705	0.595	0.883	0.015	0.555
	**Excretion**						
	**CL**	**T0.5**					
	0.877	0.616					
**Physiochemical**	**MW**	**Vol**	**Dense**	**nHA**	**nHD**	**TPSA**	**nRot**
	136.13	167.687	0.812	0	0	0	1
	**nRing**	**MaxRing**	**nHet**	**fChar**	**nRig**	**Flex**	**nStereo**
	1	6	0	0	6	0.167	0
	**LogS**	**LogD**	**LogP**				
	-2.989	2.476	3.092				
**Medicinal Chemistry**	**QED**	**Synth**	**Fsp3**	**MCE-18**	**Natural Product-likeness**	**Alarm NMR**	**BMS**
	0.518	3.113	0.6	7.5	2.528	0	0
	**Chelating**	**PAINS**	**Lipinski**	**Pfizer**	**GSK**	**Golden Triangle**	
	0	0	Accepted	Rejected	Accepted	Rejected	
**TOX 21 Pathway**	**NR-AR**	**NR-AR-LBD**	**NR-AhR**	**NR-Aromatase**	**NR-ER**	**NR-ER-LBD**	**NR-PPAR-gamma**
	0.003	0.002	0.018	0.003	0.084	0.007	0.004
	**SR-ARE**	**SR-ATAD5**	**SR-HSE**	**SR-MMP**	**SR-p53**		
	0.027	0.004	0.011	0.004	0.005		
**Toxicity**	**hERG**	**H-HT**	**DILI**	**Ames**	**ROA**	**FDAMDD**	**SkinSen**
	0.012	0.172	0.114	0.02	0.024	0.141	0.815

	**Carcinogenicity**	**EC**	**EI**	**Respiratory**			
	0.723	0.855	0.987	0.159			
**Toxicophores**	**Toxicophores**	**Acute-Aquatic Toxicity**	**LD50 oral**	**Non-Genotoxic Carcinogenicity**	**Skin Sensitization**	**SureChEMBL**	**Genotoxic Carcinogenicity Mutagenicity**
	0	1	0	0	0	0	0
	**Non-Biodegradable**						
	0						
**Environmental Toxicity**	**BCF**	**IGC50**	**LC50**	**LC50DM**			
	2.246	3.064	4.331	4.538			

Table S12: ADME, Physiochemical, Toxicity, and Environmental Toxicity of verbenone

				**Adsorption**			
**ADME**	**Pgp-inh**	**Pgp-sub**	**HIA**	**F(20%)**	**F(30%)**	**Caco-2**	**MDCK**
	0.095	0.002	0.004	0.708	0.004	-4.574	2.99E-05
		**Distribution**			**Metabolism**		
	**BBB**	**5**	**VDss**	**Fu**	**CYP1A2-inh**	**CYP1A2-sub**	**CYP2C19-inh**
	0.963	69.02%	1.595	26.52%	0.085	0.287	0.183
	**CYP2C19-sub**	**CYP2C9-inh**	**CYP2C9-sub**	**CYP2D6-inh**	**CYP2D6-sub**	**CYP3A4-inh**	**CYP3A4-sub**
	0.835	0.066	0.786	0.006	0.636	0.019	0.279
	**Excretion**						
	**CL**	**T0.5**					
	13.717	0.544					
**Physiochemical**	**MW**	**Vol**	**Dense**	**nHA**	**nHD**	**TPSA**	**nRot**

	150.1	167.921	0.894	1	0	17.07	0
	**nRing**	**MaxRing**	**nHet**	**fChar**	**nRig**	**Flex**	**nStereo**
	3	0	1	0	9	0	1
	**LogS**	**LogD**	**LogP**				
	-2.976	3.42	3.366				
**Medicinal Chemistry**	**QED**	**Synth**	**Fsp3**	**MCE-18**	**Natural Product-likeness**	**Alarm NMR**	**BMS**
	0.484	4.651	0.7	32.118	1.934	0	0
	**Chelating**	**PAINS**	**Lipinski**	**Pfizer**	**GSK**	**Golden Triangle**	
	0	0	Accepted	Accepted	Accepted	Rejected	
**TOX 21 Pathway**	**NR-AR**	**NR-AR-LBD**	**NR-AhR**	**NR-Aromatase**	**NR-ER**	**NR-ER-LBD**	**NR-PPAR-gamma**
	0.023	0.005	0.029	0.01	0.178	0.299	0.553
	**SR-ARE**	**SR-ATAD5**	**SR-HSE**	**SR-MMP**	**SR-p53**		
	0.021	0.005	0.023	0.028	0.006		
**Toxicity**	**hERG**	**H-HT**	**DILI**	**Ames**	**ROA**	**FDAMDD**	**SkinSen**
	0.004	0.128	0.377	0.015	0.389	0.207	0.591
	**Carcinogenicity**	**EC**	**EI**	**Respiratory**			
	0.622	0.975	0.977	0.961			
**Toxicophores**	**Toxicophores**	**Acute Aquatic Toxicity**	**LD50_oral**	**Non-Genotoxic Carcinogenicity**	**Skin Sensitization**	**SureChEMBL**	**Genotoxic Carcinogenicity Mutagenicity**
	0	1	0	0	1	0	0

	**Non-Biodegradable**						
	1						
**Environmental Toxicity**	**BCF**	**IGC50**	**LC50**	**LC50DM**			
	0.553	3.166	3.989	4.187			

Table S13: ADME, Physiochemical, Toxicity, and Environmental Toxicity of bornyl acetate

				**Adsorption**			
ADME	**Pgp-inh**	**Pgp-sub**	**HIA**	**F(20%)**	**F(30%)**	**Caco-2**	**MDCK**
	0.064	0	0.004	0.007	0.08	-4.552	2.36E-05
		**Distribution**			**Metabolism**		
	**BBB**	**5**	**VDss**	**Fu**	**CYP1A2-inh**	**CYP1A2-sub**	**CYP2C19-inh**
	0.798	84.17%	1.149	37.90%	0.103	0.15	0.055
	**CYP2C19-sub**	**CYP2C9-inh**	**CYP2C9-sub**	**CYP2D6-inh**	**CYP2D6-sub**	**CYP3A4-inh**	**CYP3A4-sub**
	0.904	0.108	0.754	0.007	0.594	0.11	0.245
	**Excretion**						
	**CL**	**T0.5**					
	6.063	0.243					
**Physiochemical**	**MW**	**Vol**	**Dense**	**nHA**	**nHD**	**TPSA**	**nRot**
	196.15	213.939	0.917	2	0	26.3	2
	**nRing**	**MaxRing**	**nHet**	**fChar**	**nRig**	**Flex**	**nStereo**
	2	6	2	0	9	0.222	3
	**LogS**	**LogD**	**LogP**				
	-3.388	3.128	3.263				

**Medicinal Chemistry**	**QED**	**Synth**	**Fsp3**	**MCE-18**	**Natural Product-likeness**	**Alarm NMR**	**BMS**
	0.603	4.086	0.917	39.13	2.33	0	0
	**Chelating**	**PAINS**	**Lipinski**	**Pfizer**	**GSK**	**Golden Triangle**	
	0	0	Accepted	Rejected	Accepted	Rejected	
**TOX 21 Pathway**	**NR-AR**	**NR-AR-LBD**	**NR-AhR**	**NR-Aromatase**	**NR-ER**	**NR-ER-LBD**	**NR-PPAR-gamma**
	0.727	0.022	0.007	0.011	0.169	0.203	0.28
	**SR-ARE**	**SR-ATAD5**	**SR-HSE**	**SR-MMP**	**SR-p53**		
	0.025	0.005	0.023	0.075	0.034		
**Toxicity**	**hERG**	**H-HT**	**DILI**	**Ames**	**ROA**	**FDAMDD**	**SkinSen**
	0.013	0.124	0.39	0.009	0.017	0.118	0.795
	**Carcinogenicity**	**EC**	**EI**	**Respiratory**			
	0.128	0.946	0.965	0.33			
**Toxicophores**	**Toxicophores**	**Acute Aquatic Toxicity**	**LD50_oral**	**Non-Genotoxic Carcinogenicity**	**Skin Sensitization**	**SureChEMBL**	**Genotoxic Carcinogenicity Mutagenicity**
	0	0	0	0	0	0	0
	**Non-Biodegradable**						
**Environmental Toxicity**	0						
	**BCF**	**IGC50**	**LC50**	**LC50DM**			
	2.166	3.737	4.334	4.72			

Table S14: ADME, Physiochemical, Toxicity, and Environmental Toxicity of α-phellandrene

	**Adsorption**						
	**Pgp-inh**	**Pgp-sub**	**HIA**	**F(20%)**	**F(30%)**	**Caco-2**	**MDCK**
	0.001	0.013	0.005	0.014	0.146	-4.383	2.39E-05
	**Distribution**				**Metabolism**		
	**BBB**	**5**	**VDss**	**Fu**	**CYP1A2-inh**	**CYP1A2-sub**	**CYP2C19-inh**
	0.835	92.01%	2.963	7.75%	0.258	0.471	0.178
ADME	**Metabolism**						
	**CYP2C19-sub**	**CYP2C9-inh**	**CYP2C9-sub**	**CYP2D6-inh**	**CYP2D6-sub**	**CYP3A4-inh**	**CYP3A4-sub**
	0.93	0.142	0.337	0.059	0.832	0.274	0.216
	**Excretion**						
	**CL**	**T0.5**					
	12.66	0.617					
**Physiochemical**	**MW**	**Vol**	**Dense**	**nHA**	**nHD**	**TPSA**	**nRot**
	136.13	167.687	0.812	0	0	0	2
	**nRing**	**MaxRing**	**nHet**	**fChar**	**nRig**	**Flex**	**nStereo**
	1	6	0	0	6	0.167	0
	**LogS**	**LogD**	**LogP**				
	-3.985	3.415	3.857				
**Medicinal Chemistry**	**QED**	**Synth**	**Fsp3**	**MCE-18**	**Natural Product-likeness**	**Alarm NMR**	**BMS**
	0.519	3.505	0.6	13.5	2.835	0	0
	**Chelating**	**PAINS**	**Lipinski**	**Pfizer**	**GSK**	**Golden Triangle**	
	0	0	Accepted	Rejected	Accepted	Rejected	

TOX 21 Pathway	**NR-AR**	**NR-AR-LBD**	**NR-AhR**	**NR-Aromatase**	**NR-ER**	**NR-ER-LBD**	**NR-PPAR-gamma**
	0.004	0.003	0.008	0.003	0.144	0.178	0.009
	**SR-ARE**	**SR-ATAD5**	**SR-HSE**	**SR-MMP**	**SR-p53**		
	0.151	0.004	0.177	0.037	0.006		
**Toxicity**	**hERG**	**H-HT**	**DILI**	**Ames**	**ROA**	**FDAMDD**	**SkinSen**
	0.024	0.76	0.014	0.011	0.038	0.723	0.417
	**Carcinogenicity**	**EC**	**EI**	**Respiratory**			
	0.344	0.183	0.957	0.855			
**Toxicophores**	**Toxicophores**	**Acute Aquatic Toxicity**	**LD50_oral**	**Non-Genotoxic Carcinogenicity**	**Skin Sensitization**	**SureChEMBL**	**Genotoxic Carcinogenicity Mutagenicity**
	0	1	0	0	0	0	0
	**Non-Biodegradable**						
**Environmental Toxicity**	0						
	**BCF**	**IGC50**	**LC50**	**LC50DM**			
	2.36	3.08	3.674	4.176			

Table S15: ADME, Physiochemical, Toxicity, and Environmental Toxicity of sabinene

				**Adsorption**			
ADME	**Pgp-inh**	**Pgp-sub**	**HIA**	**F(20%)**	**F(30%)**	**Caco-2**	**MDCK**
	0	0	0.003	0.304	0.02	-4.4	1.75E-05
		**Distribution**				**Metabolism**	
	**BBB**	**5**	**VDss**	**Fu**	**CYP1A2-inh**	**CYP1A2-sub**	**CYP2C19-inh**
	0.976	69.45%	1.169	29.20%	0.497	0.384	0.123
				**Metabolism**			

	CYP2C19-sub	CYP2C9-inh	CYP2C9-sub	CYP2D6-inh	CYP2D6-sub	CYP3A4-inh	CYP3A4-sub
	0.9	0.113	0.398	0.045	0.744	0.108	0.569
	Excretion						
	CL	T0.5					
	11.198	0.194					
Physiochemical	MW	Vol	Dense	nHA	nHD	TPSA	nRot
	136.13	161.767	0.842	0	0	0	1
	nRing	MaxRing	nHet	fChar	nRig	Flex	nStereo
	2	6	0	0	8	0.125	2
	LogS	LogD	LogP				
	-3.657	3.483	3.274				
Medicinal Chemistry	QED	Synth	Fsp3	MCE-18	Natural Product-likeness	Alarm NMR	BMS
	0.486	4.343	0.8	30.667	3.303	0	0
	Chelating	PAINS	Lipinski	Pfizer	GSK	Golden Triangle	
	0	0	Accepted	Rejected	Accepted	Rejected	
TOX 21 Pathway	NR-AR	NR-AR-LBD	NR-AhR	NR-Aromatase	NR-ER	NR-ER-LBD	NR-PPAR-gamma
	0.019	0.003	0.004	0.008	0.081	0.16	0.003
	SR-ARE	SR-ATAD5	SR-HSE	SR-MMP	SR-p53		
	0.022	0.005	0.034	0.017	0.009		
Toxicity	hERG	H-HT	DILI	Ames	ROA	FDAMDD	SkinSen
	0.009	0.119	0.155	0.029	0.088	0.156	0.059
	Carcinogenicity	EC	EI	Respiratory			
	0.305	0.189	0.976	0.305			

	Toxicophores	Acute Aquatic Toxicity	LD50_oral	Non-Genotoxic Carcinogenicity	Skin Sensitization	SureChEMBL	Genotoxic Carcinogenicity Mutagenicity
Toxicophores	0	1	0	0	0	0	0
	Non-Biodegradable						
	0						
Environmental Toxicity	BCF	IGC50	LC50	LC50DM			
	2.874	3.776	4.337	4.4			

Table S16: ADME, Physiochemical, Toxicity, and Environmental Toxicity of p-cymene

				Adsorption			
	Pgp-inh	Pgp-sub	HIA	F(20%)	F(30%)	Caco-2	MDCK
	0.011	0.005	0.004	0.211	0.931	-4.302	1.96E-05
		Distribution				Metabolism	
	BBB	5	VDss	Fu	CYP1A2-inh	CYP1A2-sub	CYP2C19-inh
	0.728	94.38%	2.139	6.09%	0.941	0.944	0.855
ADME				Metabolism			
	CYP2C19-sub	CYP2C9-inh	CYP2C9-sub	CYP2D6-inh	CYP2D6-sub	CYP3A4-inh	CYP3A4-sub
	0.864	0.574	0.61	0.778	0.755	0.084	0.62
	Excretion						
	CL	T0.5					
	7.38	0.276					
Physiochemical	MW	Vol	Dense	nHA	nHD	TPSA	nRot
	134.11	165.05	0.813	0	0	0	1
	nRing	MaxRing	nHet	fChar	nRig	Flex	nStereo
	1	6	0	0	6	0.167	0

	LogS	LogD	LogP				
	-3.919	3.778	3.994				
Medicinal Chemistry	QED	Synth	Fsp3	MCE-18	Natural Product-likeness	Alarm NMR	BMS
	0.553	1.251	0.4	6	-0.717	0	0
	Chelating	PAINS	Lipinski	Pfizer	GSK	Golden Triangle	
	0	0	Accepted	Rejected	Accepted	Rejected	
TOX 21 Pathway	NR-AR	NR-AR-LBD	NR-AhR	NR-Aromatase	NR-ER	NR-ER-LBD	NR-PPAR-gamma
	2.386	3.641	4.072	4.199	2.386	3.641	4.072
	SR-ARE	SR-ATAD5	SR-HSE	SR-MMP	SR-p53		
	0.015	0.004	0.014	0.007	0.001		
Toxicity	hERG	H-HT	DILI	Ames	ROA	FDAMDD	SkinSen
	0.031	0.037	0.201	0.018	0.079	0.048	0.159
	Carcinogenicity	EC	EI	Respiratory			
	0.386	0.958	0.993	0.03			
Toxicophores	Toxicophores	Acute Aquatic Toxicity	LD50_oral	Non-Genotoxic Carcinogenicity	Skin Sensitization	SureChEMBL	Genotoxic Carcinogenicity Mutagenicity
	0	1	0	0	0	0	0
	Non-Biodegradable						
	0						
Environmental Toxicity	BCF	IGC50	LC50	LC50DM			
	2.874	3.776	4.337	4.4			
