# Supplementary figures and images for: Vitex negundo L. Essential Oil: Odorant Binding Protein Efficiency Using Molecular Docking Approach and Studies of the Mosquito Repellent
Source: Insects. 2021 Nov 26;12(12):1061. doi: 10.3390/insects12121061 (PMC8703855; doi:10.3390/insects12121061)

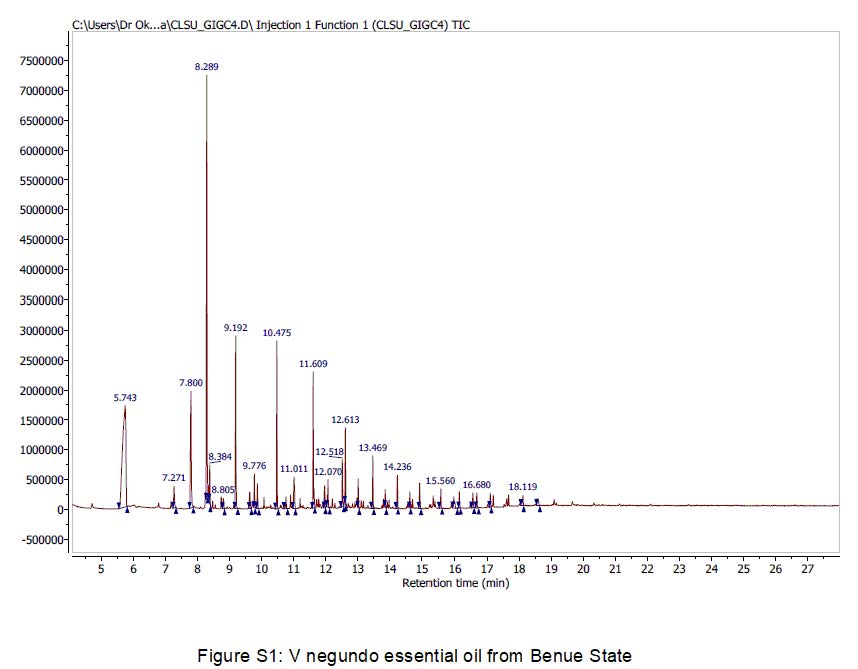

Supplement: Supplementary file 1 [file insects-12-01061-s001.zip › Figure S1.jpg]

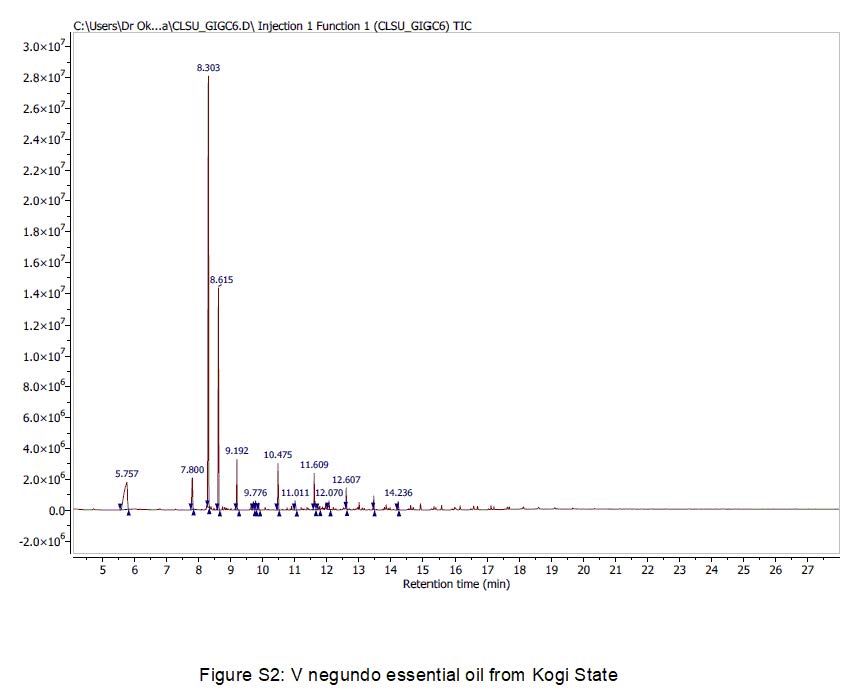

Supplement: Supplementary file 1 [file insects-12-01061-s001.zip › Figure S2.jpg]

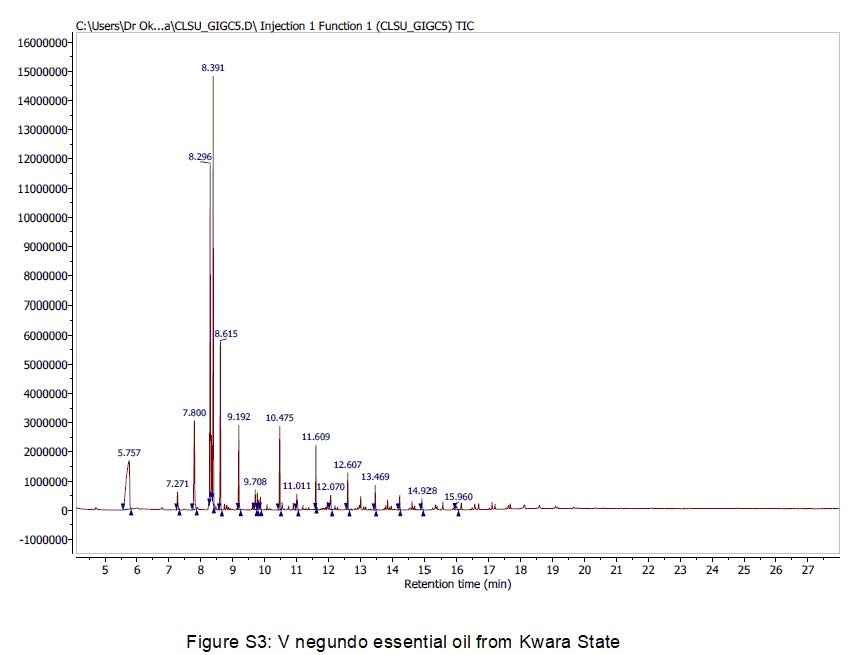

Supplement: Supplementary file 1 [file insects-12-01061-s001.zip › Figure S3.jpg]

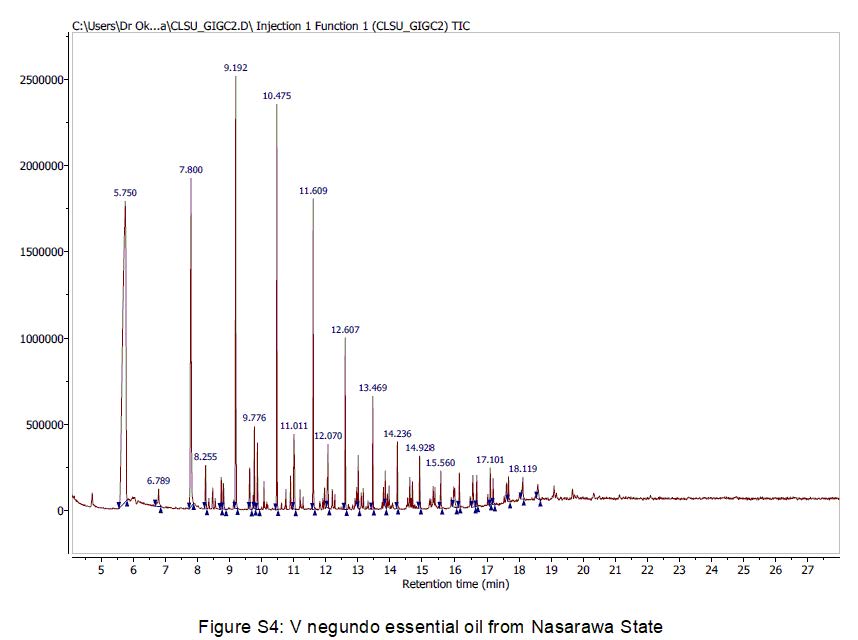

Supplement: Supplementary file 1 [file insects-12-01061-s001.zip › Figure S4.jpg]

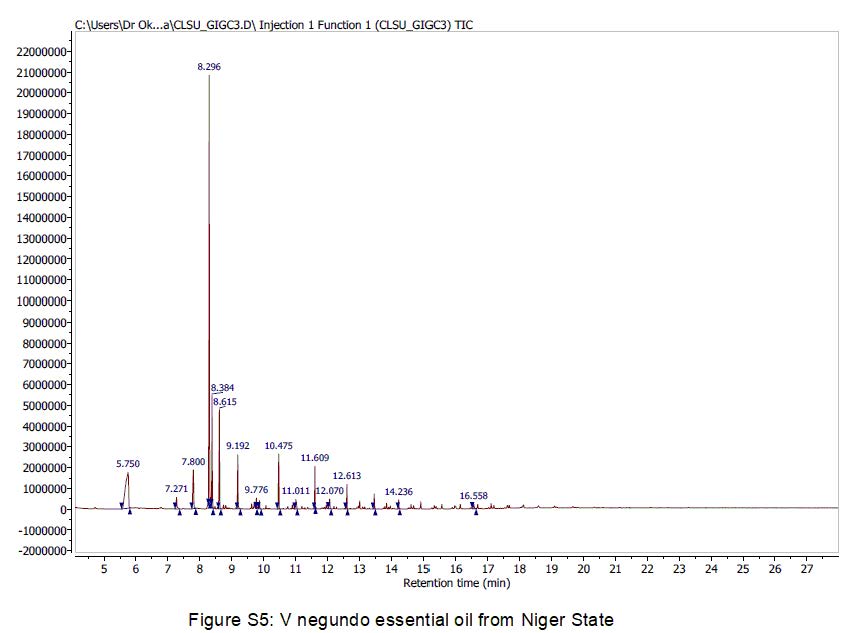

Supplement: Supplementary file 1 [file insects-12-01061-s001.zip › Figure S5.jpg]

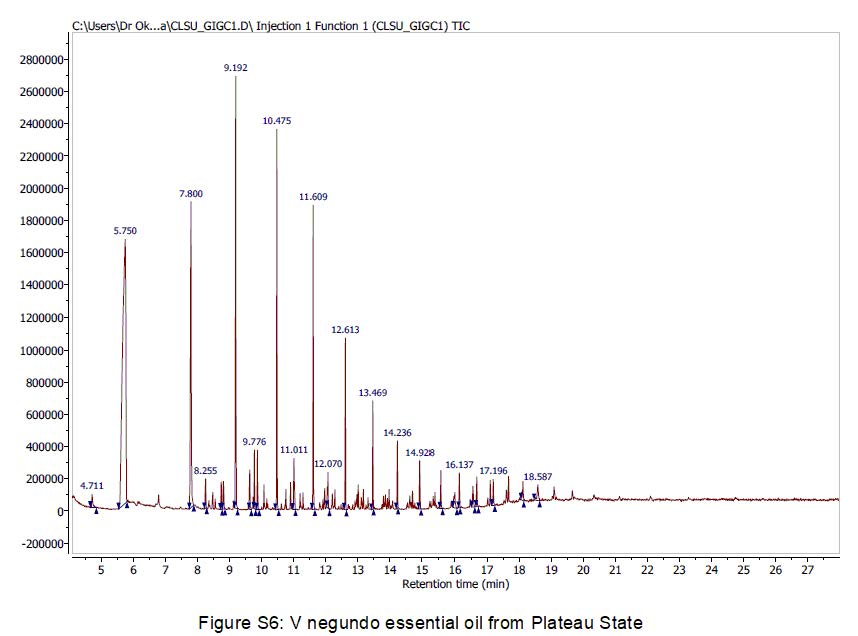

Supplement: Supplementary file 1 [file insects-12-01061-s001.zip › Figure S6.jpg]
